# Synergistic Machine Learning Guided Discovery of ABa_3_(BSe_3_)_2_X (A = Rb, Cs; X = Cl, Br, I): A Promising Family as Property‐Balanced IR Functional Materials

**DOI:** 10.1002/advs.202417851

**Published:** 2025-04-26

**Authors:** Yihan Yun, Mengfan Wu, Zhihua Yang, Guangmao Li, Shilie Pan

**Affiliations:** ^1^ Research Center for Crystal Materials; State Key Laboratory of Functional Materials and Devices for Special Environmental Conditions; Xinjiang Key Laboratory of Functional Crystal Materials; Xinjiang Technical Institute of Physics and Chemistry Chinese Academy of Sciences 40–1 South Beijing Road Urumqi 830011 China; ^2^ Center of Materials Science and Optoelectronics Engineering University of Chinese Academy of Sciences Beijing 100049 China

**Keywords:** chalcogenides, data‐driven machine learning, nonlinear optical materials, selenoborates

## Abstract

Discovering novel infrared functional materials (IRFMs) hold tremendous significance for laser industry. Incorporating artificial intelligence into material discovery has been recognized as a pivotal trend driving advancements in materials science. In this work, an IRFM predictor based on machine learning (ML) is developed for the pre‐selection of the most promising candidates, in which interpretable analyses reveal the prior domain knowledge of IRFMs. Under the guidance of this IRFM predictor, a series of selenoborates, ABa_3_(BSe_3_)_2_X (A = Rb, Cs; X = Cl, Br, I) are successfully predicted and synthesized. Comprehensive characterizations together with first‐principles analyses reveal that these materials exhibit preferred properties of wide bandgaps (2.92 – 3.04 eV), moderate birefringence (0.145 – 0.170 at 1064 nm), high laser‐induced damage thresholds (LIDTs) (4 – 6 Ý AGS) and large second harmonic generation (SHG) responses (0.9 – 1 × AGS). Structure‐property relationship analyses indicate that the [BSe_3_] unit can be regarded as a potential gene for exploring novel IRFMs. This work may open an avenue for exploring high‐performance materials.

## Graphical abstract

A synergistic framework for interpretable ML‐assisted target discovery of selenoborates is developed. ABa_3_(BSe_3_)_2_X (A = Rb, Cs; X = Cl, Br, I) are successfully predicted and synthesized, exhibiting significant potential to be promising IR functional materials. This work promotes the cooperation of ML technology and materials science.

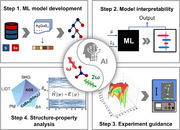

## 1 Introduction

With the increasing demand on applications of the laser technology,^[^ ^1^, ^2^, ^3^ ^]^ optical devices need development accordingly,^[^ ^4^, ^5^, ^6^, ^7^, ^8^ ^]^ such as electro‐optic modulation, deflection, Q‐switch and photorefractive devices.^[^ ^9^, ^10^ ^]^ At present, researches on photoelectric functional crystal materials have been worldwide focus in materials science.^[^ ^11^, ^12^, ^13^, ^14^, ^15^, ^16^, ^17^, ^18^, ^19^, ^20^, ^21^, ^22^, ^23^, ^24^, ^25^, ^26^, ^27^ ^]^ Infrared functional materials (IRFMs) encompass a broad range of material types, such as IR birefringent materials, nonlinear optical (NLO) materials, and fiber‐optical materials, all of which are essential in the laser industry. Among them, IR NLO materials, serving as the core components of tunable infrared laser systems, have garnered widespread attention. Nowadays, the available IR NLO materials are limited, namely commercial crystals AgGaS_2_ (AGS), AgGaSe_2_ (AGSe), and ZnGeP_2_ (ZGP).^[^ ^28^, ^29^, ^30^, ^31^ ^]^ Nevertheless, serious two‐photon absorption under common pump light sources (≈1 µm) and low LIDT, etc. hindered their further applications. Consequently, there is a pressing demand to investigate novel materials as candidates for the development of IR laser applications.

During recent decades, several IRFMs were obtained as quaternary chalcogenides, mixed metal halides, phosphides, etc.^[^ ^32^, ^33^, ^34^, ^35^, ^36^, ^37^, ^38^, ^39^, ^40^, ^41^, ^42^, ^43^, ^44^, ^45^, ^46^, ^47^, ^48^, ^49^, ^50^, ^51^, ^52^ ^]^ While, the long discovery process by traditional trial‐and‐error method was time‐consuming and laborious. As a result, few materials could fulfill the perfect balance among the bandgap, laser‐induced damage threshold (LIDT), birefringence, and second harmonic generation (SHG) responses. To address this limitation, it is necessary to explore new approaches that can lessen labor efforts, besides shorten the exploration process of high‐performance IRFMs. With the development of artificial intelligence, data‐driven machine learning (ML) technology has become a powerful tool for designing and discovering advanced materials.^[^ ^53^, ^54^, ^55^, ^56^, ^57^, ^58^, ^59^, ^60^, ^61^, ^62^ ^]^ ML‐based high‐throughput predictions for a large amount of materials can be orders of magnitude faster than experiments, allowing them to be used to quickly understand trends in materials properties and accelerate materials discovery, such as using inference and global optimization to find memory alloys with low thermal hysteresis, adapting extra trees algorithm to predict the thermodynamic stability of perovskite oxides, and establishing an autonomous laboratory for solid‐state synthesis, discovering several novel oxides and phosphates.^[^ ^63^, ^64^, ^65^, ^66^, ^67^ ^]^ These ML models have demonstrated strong performance within their respective material systems. Therefore, it is expected that ML has significant potential in accelerating the discovery of novel IRFMs.

A proper application system is prerequisite for discovering new high‐performance material based on ML. Given that chalcogenides form a rich potential class of IRFMs, establishing efficient property prediction models in chalcogenide system may rapidly identify the most promising candidates for laboratory synthesis. As a branch of chalcogenides, selenoborates may exhibit wider IR transmittance and larger SHG response than isostructural thioborates.^[^ ^68^, ^69^, ^70^, ^71^ ^]^ Besides, microscopic unit calculations performed using Gaussian09^[^ ^72^ ^]^ reveal the [BSe_3_] unit exhibits the highest polarizability anisotropy and hyperpolarizability, suggesting its potential for achieving significant birefringence and SHG (Figure S1, Supporting Information). Introducing strong ionic ions into the selenoborate covalent structure may result in a larger band gap and affect the arrangement of covalent units.^[^ ^73^ ^]^ Meanwhile, heavy elements are more conducive to obtaining a wide IR transmission range. Thus, A‐AE‐B_x_Se_y_‐X (A = Rb, Cs; AE = Ba; X = Cl, Br, I) system is a potential system for exploring IRFMs with balanced properties.

In this work, we developed a synergistic framework for interpretable ML‐assisted target synthesis of property‐balanced IRFMs. Herein, the developed IRFM predictor enables the pre‐selection of chemical compositions with a high likelihood of being synthesized and suitable bandgaps, prior to the time‐consuming experimental process. Specially, the interpretable mechanisms of the “black‐box” model were finely analyzed. Combining the guidance of this predictor and experimental synthesis, a series of novel IRFMs ABa_3_(BSe_3_)_2_X (A = Rb, Cs; X = Cl, Br, I), which are the first reported selenoborate halides (Table S1, Supporting Information) were obtained, showing excellent properties as IR birefringent/NLO materials, namely wide bandgaps (2.92–3.04 eV), moderate birefringence (0.145–0.170 at 1064 nm), high LIDTs (4‐6 × AGS) and large SHG responses (0.9‐1 × AGS). Structure‐property analyses based on first‐principles theory proved that the π‐conjugated [BSe_3_] is the origin of wide bandgaps, large birefringence, and high SHG responses, which reveals that [BSe_3_] unit is a potential gene for exploring novel IRFMs. This marks the first successful case of interpretable ML technology in assisting the experimental synthesis of IRFMs. This work will open the door to designing high‐performance IRFMs and promote interdisciplinary progress in ML and new material research.

## 2 Results and Discussion

### 2.1 Synergistic Framework for ML‐Assisted Target Synthesis of IRFMs

**Figure 1:**
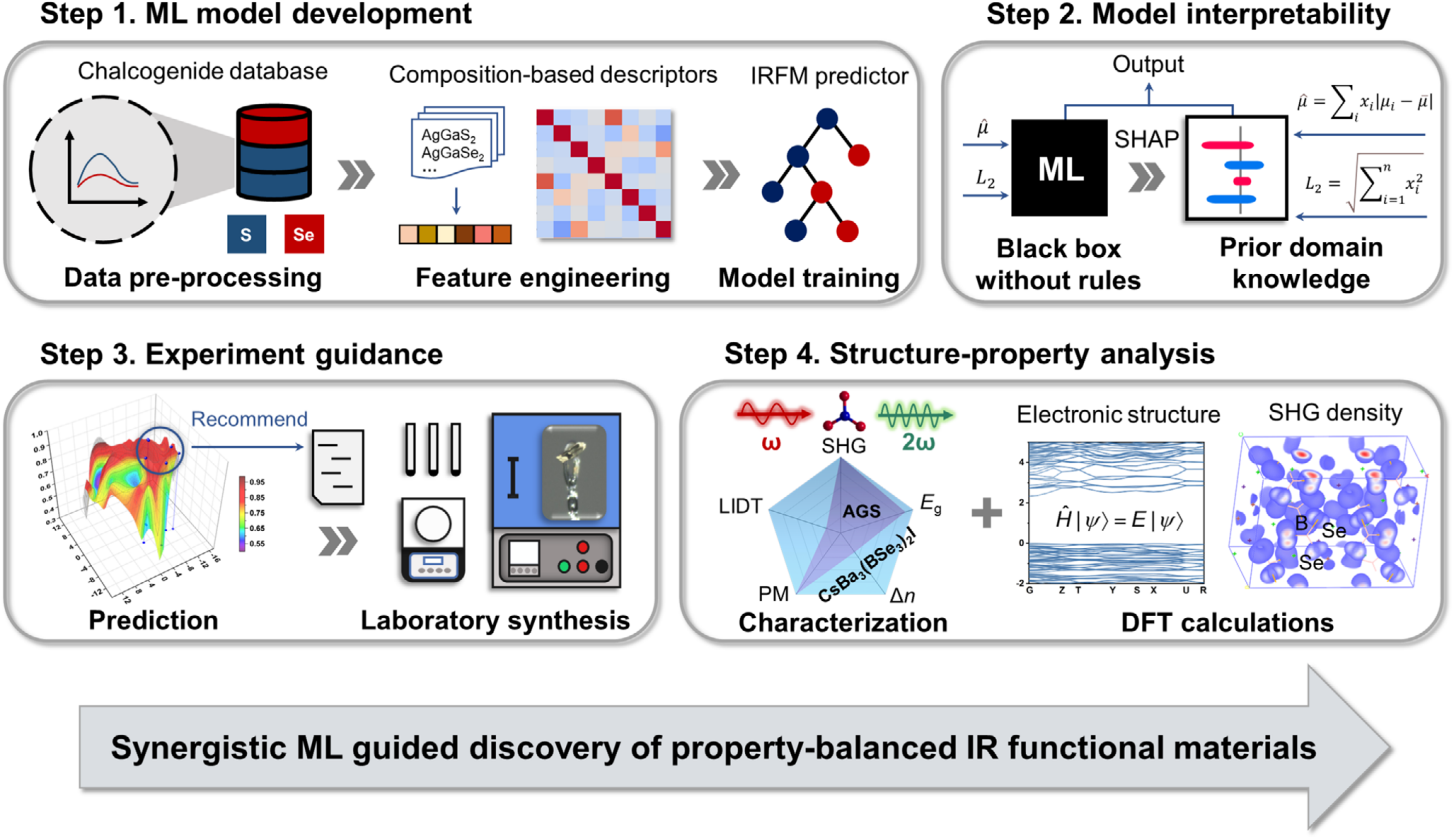
Schematic of the synergistic framework for interpretable ML‐assisted target synthesis of IRFMs.

Our proposed synergistic framework for ML‐assisted target discovery of IRFMs contains four steps, namely ML model development, model interpretability, experiment guidance, and structure‐property analysis (**Figure** **1**). First, feature engineering was performed on the database, converting the chemical formulas of these chalcogenides into fixed‐length vectors. With these vectors, a IRFM predictor based on ML classification models was then developed to predict the synthesis feasibility (SF) and bandgap (*E*_g_) of chalcogenides. Second, we conducted interpretable analysis on the optimal models to uncover the mechanisms how different descriptors influenced the SF and *E*_g_, thereby obtaining the prior domain knowledge of IRFMs. The third step is applying this IRFM predictor in the selenoborate system to make efficient predictions, enabling the rapid identification of the most promising chemical compositions with a high likelihood of being synthesized and possessing suitable bandgaps. The recommended compounds were targeted in laboratory synthesis, leading to the successful synthesis of a series of novel IRFMs. Ultimately, we performed experimental performance characterization and density functional theory (DFT) calculations for these newly synthesized IRFMs to reveal the in‐depth structure‐property relationship. This framework provides a guidance for the targeted synthesis of chalcogenides and marks the first successful integration of interpretable ML with experimental synthesis in this field, leading to the discovery of novel promising IRFMs.

### 2.2 Developed IRFM predictor for Predicting Synthesis Feasibility and Bandgaps of Chalcogenides

An IRFM predictor for efficiently providing the most promising chemical compositions for experiment was developed, comprising two ML classifiers designed to predict the SF and *E*_g_ of chalcogenides. As we know, an appropriate input dataset is one of the key factors in building ML models with strong generalization ability. In this work, the input entries were chemical compositions with common chalcogen elements in IRFMs, the formation enthalpies above thermodynamical convex hull (*E*_hull_) and *E*_g_ of the corresponding materials. Herein, 154718 entries from the Materials Project (MP) database^[^ ^74^ ^]^ via application programming interface (API) were retrieved. First, we carefully pre‐processed the MP database to refine the selection: polynary compounds containing chalcogen elements (S or Se) were selected, while elements such as rare gases, lanthanides, and actinides were excluded. Second, SF was assessed using the formation enthalpies above thermodynamical convex hull (*E*_hull_) as the first indicator, which quantifies the decomposition energy of a compound into stable phases on the phase diagram. For compounds with identical compositions but different phases, we selected the entry with the lowest *E*_hull_. As a result, a chalcogenide database that encompasses a total of 5451 chemical compositions was obtained, covering a broad range of chemical environments with diverse cationic elements (alkali metals, alkaline earth metals, and transition metals) and various anionic units (Figures S2 and S3, Supporting Information), which reflects the representativeness of this dataset. Prior research indicating that over 80% of materials in the Inorganic Crystal Structure Database have *E*_hull_ values below 36 meV atom^−1^,^[^ ^75^ ^]^ this threshold was adopted to establish the first ML classifier. Thereby, compounds not exceeding the threshold of 36 meV atom^−1^ are considered to possess a higher likelihood of being easily synthesized in experiment and are labeled as ‘1′ (High‐SF), conversely, those exceeding the threshold are labeled as ‘0′ (Low‐SF). Third, a bandgap classification threshold of 1.5 eV was set based on the Perdew–Burke–Ernzerhof (PBE) bandgaps in the database as the second indicator.^[^ ^76^, ^77^, ^78^ ^]^ 2430 compounds with high‐SF and non‐zero *E*_g_ constitute the second database were used to train the second ML classifier for predicting *E*_g_. Moreover, compounds with *E*_g_ higher than 1.5 eV are labeled as ‘1′ (High‐*E*_g_), while others are labeled as ‘0′ (Low‐*E*_g_).

Further, we adopted the Magpie method^[^ ^79^ ^]^ via the Matminer package to generate 149 dimensional digital descriptors for each chemical composition, employing modules such as Stoichiometry, ElementProperty, ValenceOrbital, IonProperty and AtomicOrbitals.^[^ ^80^, ^81^ ^]^ These descriptors capture intrinsic properties of the elements and their proportions in chemical compositions, serving as input for ML models. This featurization process does not require time‐consuming DFT calculations, thereby enabling efficient screening across the broad chemical space in subsequent applications. Taking the training process for predicting SF as an example, nine widely used classification algorithms were adopted to select the optimal model based on Scikit‐learn package (details are shown in experimental section).^[^ ^82^ ^]^ The Area Under the Receiver Operating Characteristic (ROC) Curve (AUC) and accuracy indicate that LightGBM balanced both metrics, making it the optimal choice for the SF classification task (Figure S4, Supporting Information). In order to avoid the curse of dimensionality, model‐based wrapper method and Pearson correlation coefficient (*ρ*) method were adopted.^[^ ^83^ ^]^ The curve of AUC values from ten‐fold cross validation, varying with the number of features, indicates that the model reaches its optimal performance and remains stable when the number of features reaches 74 (Figure S5, Supporting Information). Subsequently, the Pearson correlation coefficient of the selected 74 features was calculated, which is defined as *ρ =* cov(*x_i_*, *x_j_*) / σ(*x_i_*)σ(*x_j_*), where cov(*x*_i_, *x*_j_) and σ(*x_i_*_/_ *_j_*) are the covariance of features *x_i_* and *x_j_* and standard deviation of the feature *x_i_*_/_ *_j_*, respectively. For two related features with |*ρ*| > 0.9, the one with higher importance will be retained. As a result, 55 important features were retained as the optimal feature set (Figure S6 and Table S2, Supporting Information). Hyperparameters of LightGBM model in this work were tuned based on Bayesian optimization as implemented in the Optuna package,^[^ ^84^ ^]^ and the optimal hyperparameters were listed in Table S3 (Supporting Information). In this manner, the second classifier for predicting *E_g_* was trained using the XGBoost algorithm, which showed excellent performance and robustness against overfitting. The corresponding selected features and optimal hyperparameters are listed in Tables S4 and S5 (Supporting Information).

**Figure 2:**
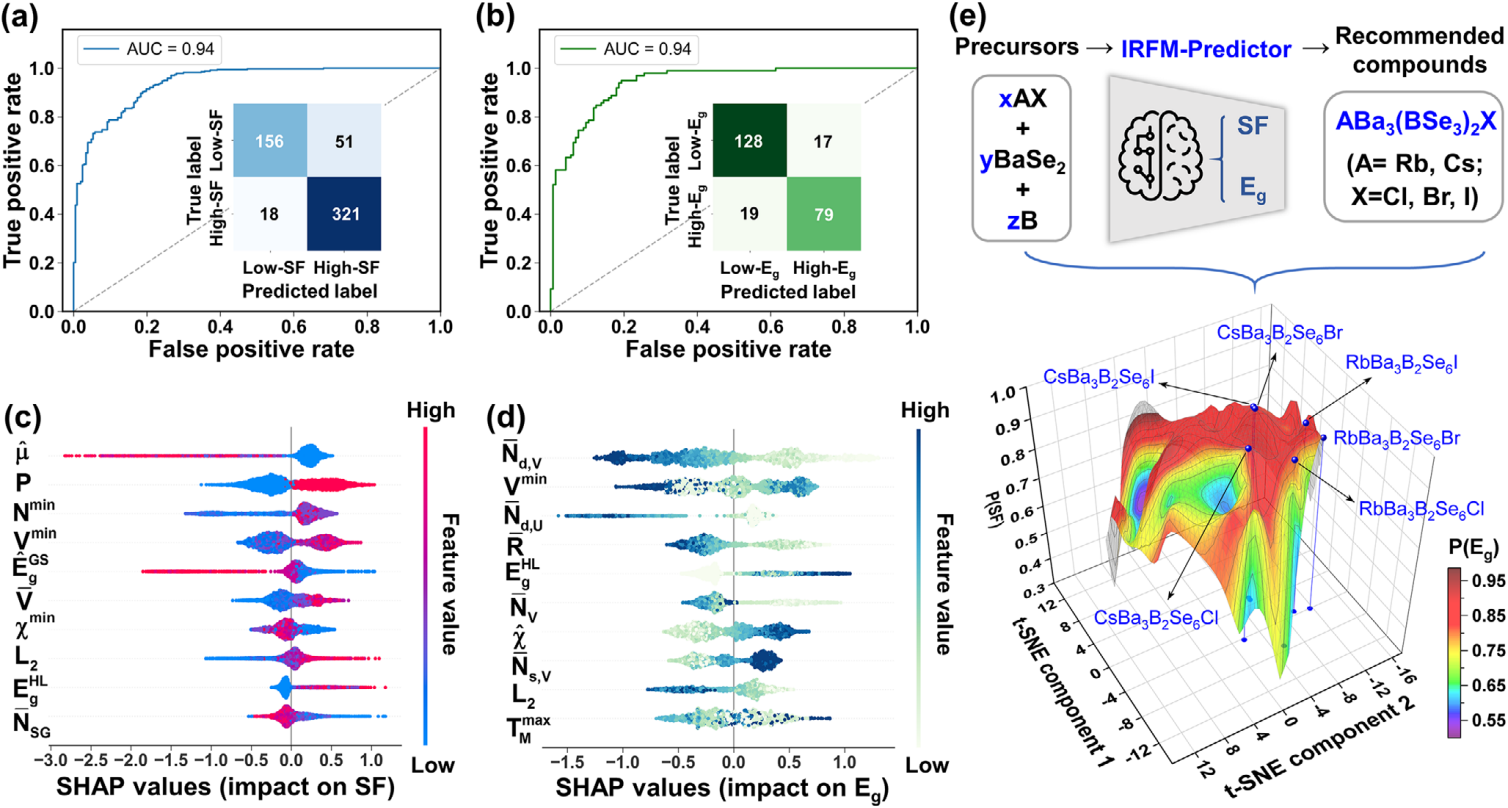
ML model development and interpretability. a,b) ROC curves and confusion matrixs of the two ML classifiers for predicting SF based on LightGBM and *E*_g_ based on XGBoost; c,d) SHAP feature importance plots for revealing the impact of different features on SF and *E*_g_; e) Constructed 3D property surface of the (AX)_x_(BaSe_2_)_y_(B)_z_ system based on the ML‐predicted P(SF) and P(*E*_g_) using t‐SNE visualization.

Finally, we evaluated the generalization ability of these two ML classifiers for predicting SF and *E*_g_ based on the randomly split training set (90% of the input dataset) and test set (10% of the input dataset). The results reveal that the test set in both classifiers achieve high performance with the ROC AUC of 0.94 (**Figures** **2**a,b). Thus, this pre‐trained IRFM predictor is considered to be reliable and applied to further guide the experimental synthesis of IRFMs in the unexplored chemical space.

### 2.3 Prior Domain Knowledge for Chalcogenides Based on Model Interpretability

Owing to the “black‐box” nature of the ML models, the knowledge of guiding principles is commonly scarce. In this work, the SHapley Additive exPlanations (SHAP)^[^ ^85^, ^86^ ^]^ method based on game theory was used to explain the effect of each feature on SF and *E*_g_. As a result, several prior domain knowledge for chalcogenide systems was inferred and summarized through the analysis of the physical meanings of these features. Figures 2c,d show the top 10 features according to their impact on SF and *E*_g_ with the horizontal axes representing the SHAP values. For the LightGBM model employed to predict SF, the most important feature is $\hat{\mu }$, which is defined as: $\hat{\mu }=\sum_{i}x_{i}\left|\mu_{i}-\bar{\mu }\right|\;$, where µ_*i*_ means the DFT magnetic moment of T = 0 K ground state for element *i* in a compound, and *x_i_* means the atomic fraction. The smaller $\hat{\mu }$ value, the more positive effect model gained. For atomic structures with multiple unpaired electrons, these unpaired electrons generate magnetic moments based on their spin (*µ*_*s*_) and orbital motion (*µ*_*e*_). The interaction between *µ*_*s*_ and *µ*_*e*_ forms the total magnetic moment of an atom. In terms of dataset elements, Sc, Ti, Mn, Fe, Co, Ni and rare earth atoms have multiple unpaired electrons. Compounds containing these elements tend to have low synthesis feasibility. *P* is a Boolean denoting whether it is possible to form a neutral ionic compound assuming each element takes exactly one of its common charge states. The true value of *P* has a positive effect on the model output, which means that elements with strong ionicity are more likely to form stable structures. *N* ^min^ means the atomic number of the lightest atom in a compound. The smaller *N* ^min^ value, the more negative effect model gained, namely compounds containing lighter atoms are more difficult to form stable structures. *V* ^min^ represents the smallest atom volume in a compound. Ordinarily, the volume of an atom (*V*) is mainly determined by the outer electrons, and the number of electron layers directly affects the radius of the atom (*r*). Their relationship could be expressed as: *V* ∝*r*. As for the main group elements of the same period from heavier to lighter and the same main group from top to bottom, the *r* and *V* values gradually increase. Notably, transition elements have smaller *V* value than IA and IIA elements, making it more difficult for chalcogenides containing transition metal elements as the smallest atomic volume to form stable structures.

As for the XGBoost model for predicting *E*_g_, ${\bar{N}}_{d,V}$ exhibits the greatest impact, representing the mean number of filled *d* valence orbitals. When a compound exhibits a smaller ${\bar{N}}_{d,V}$, it is associated with a positive SHAP value. In other words, the lower number of filled *d* valence orbitals of the constituent elements in a compound, the more favorable it is to achieve a larger bandgap. An interesting phenomenon is that excessively large or small *V* ^min^ values are unfavorable for achieving a wide bandgap. Instead, selecting elements with moderate *V* ^min^ is essential for obtaining larger bandgaps. For the feature ${\bar{N}}_{d,U}$, which is defined as the mean number of unfilled *d* valence orbitals. Similar to ${\bar{N}}_{d,V}$, a smaller ${\bar{N}}_{d,U}\;$ is also more conducive to achieving a larger *E*_g_. This is because the number of unoccupied orbitals is denoted as 0 if shell unoccupied, which also indicates a preference for elements without *d* valence orbitals. This observation also explains the experimental preference for incorporating alkali and alkaline earth metals as cations. $\bar{R}$, representing the mean periodic table row among elements in a composition, shows a positive effect on *E*_g_ with its lower values. This suggests that selecting elements from earlier rows of the periodic table is more likely to result in larger *E*_g_ values.

We also note that some features, such as *V* ^min^ and *L*_2_, have significant effects on both SF and *E*_g_. The feature *L*_2_ captures the fraction of the elements presented in compounds, and it is not affected by what these elements are. *L*_2_ is defined as: $L_{2}=\sqrt{\sum_{i=1}^{n}x_{i}^{2}}\;$ ($x_{i}>0,\;x_{1}+x_{2}+\cdots +\;x_{n}=\;1$), where *x_i_* means the atomic fraction. *L*_2_ exhibits a opposite trend in its effect on SF and *E*_g_, i.e., while larger *L*_2_ values facilitate experimental synthesis, they do not favor the formation of larger bandgaps. Therefore, a balanced selection of moderate *L*_2_ values is required in experiments to balance these two factors. We can infer that when *n* = 1, *L*_2_ reaches its maximum value. This indicates that compared to *n*‐membered compounds, simple substance of an element has a greater possibility of stable existence. In *n*‐membered chalcogenides, it is not easy to form a stable structure when the proportion of each element is the same. Figure S7 (Supporting Information) shows the specific effects of the feature *L*_2_ both on SF and *E*_g_, which recommends a preferred region of 0.54 ≤ *L*_2_ ≤ 0.65. This region can serve as a priori guidance for experimental researchers in designing composition ratios, aiming to enhance the likelihood of synthesizing stable IRFMs with wide bandgaps.

### 2.4 Guided Synthesis in Selenoborate Systems as IRFMs via ML‐Prediction

Benefited from the above work, we applied the pre‐trained IRFM predictor into predicting unexplored selenoborate systems to guide experimental synthesis. Based on the prior domain knowledge from interpretable analysis, choosing heavier alkali and alkaline earth metals as cations and strong ionic halogens as anions is beneficial for obtaining suitable $\hat{\mu }$, *N* ^min^, *P*, and ${\bar{N}}_{d,V}$ value, further higher SF and wider *E*_g_. Microscopic unit calculations already indicated that the [BSe_3_] unit exhibits the highest polarizability anisotropy and hyperpolarizability, selenoborates have great potential as high performance IRFMs (Figure S1, Supporting Information). So we restricted cations to Rb^+^, Cs^+^, and Ba^2+^ while introducing strong ionic ions Cl^−^, Br^−^, and I^−^ into the selenoborate covalent structure to potentially increase the bandgap, influence covalent unit arrangement.^[^ ^87^ ^]^ As a result, we chose the (AX)_x_(BaSe_2_)_y_(B)_z_ system (A = Rb, Cs; X = Cl, Br, I; x, y, z ∈ [1,3]) as the unexplored chemical space in experiment based on the three precursors: AX, BaSe_2_, and B. This chemical space offers 162 possible ratios, which clearly makes it highly time‐consuming and labor‐intensive to test one by one in experiment. At this point, the significant advantage of ML becomes obviously evident, allowing us to rapidly evaluate the most promising candidates among these compositions to prioritize for experimental investigation within seconds. As a result, we construct the 3D property surface of the (AX)_x_(BaSe_2_)_y_(B)_z_ system based on the predicted P(SF) (the predicting probability for high‐SF) and P(*E*_g_) (the predicting probability for high‐*E_g_*) using the IRFM predictor, which is visualized via the t‐distributed stochastic neighbor embedding (t‐SNE) method as shown in Figure 2e. In the predicted set of highly composable element ratio (be represented by (x, y, z)), (1, 3, 2) ratio frequently appear, which not only exhibits the highest P(SF) among several ratios, but also demonstrates a larger P(*E*_g_) (Figure S8, Supporting Information). This suggests that ABa_3_(BSe_3_)_2_X family is a promising system, with a high likelihood of being synthesized in experiments and a strong potential for wide bandgaps.

Inspiringly, utilizing a spontaneous crystallization method, we successfully synthesized ABa_3_(BSe_3_)_2_X (A = Rb, Cs; X = Cl, Br, I) base on the pre‐selected promising (1,3,2) ratio via ML, employing carbon‐coated quartz tubes and graphite crucibles to prevent B‐Si substitution reactions at high temperatures.^[^ ^88^, ^89^, ^90^ ^]^ The structures were refined by Olex2 and their atomic coordinates, isotropic displacement parameters, bond lengths, angles and bond valence sum (BVS) are detailed in Tables S6–S26 (Supporting Information). Among them, there are five compounds crystallizing in noncentrosymmetric (NCS) space groups *Cmc*2_1_ and one compound crystallizes in the centrosymmetric (CS) space group *Pbca* (**Table** **1**).

**Table 1:** Selected information of crystal data and structure refinement for ABa_3_(BSe_3_)_2_X (A = Rb, Cs; X = Cl, Br, I).

Empirical formula	RbBa_3_(BSe_3_)_2_Cl	CsBa_3_(BSe_3_)_2_Cl
Formula weight	1028.32	1075.76
Temperature	245.0 K	245.0 K
Crystal system, space group	Orthorhombic, *Cmc*2_1_	Orthorhombic, *Pbca*
Unit cell dimensions	*a* = 15.403(2) Å	*a* = 11.8928(8) Å
	*b* = 11.8262(18) Å	*b* = 8.6874(5) Å
	*c* = 8.6681(14) Å	*c* = 31.030(2) Å
Volume	2551.80(18) Å^3^	3206.0(4) Å^3^
*Z*, calculated density	4, 4.326 g/cm^3^	8, 4.458 g/cm^3^
Flack parameter	0.00(5)	/
Empirical formula	RbBa_3_(BSe_3_)_2_Br	CsBa_3_(BSe_3_)_2_Br
Formula weight	1072.78	1120.22
Temperature	100.0 K	245.0 K
Crystal system, space group	Orthorhombic, *Cmc*2_1_	Orthorhombic, *Cmc*2_1_
Unit cell dimensions	*a* = 16.0147(11) Å	*a* = 16.1046(13) Å
	*b* = 11.6688(7) Å	*b* = 11.7634(10) Å
	*c* = 8.5898(5) Å	*c* = 8.5819(6) Å
Volume	1605.20(17) Å^3^	1625.8(2) Å^3^
*Z*, calculated density	4, 4.439 g/cm^3^	4, 4.577 g/cm^3^
Flack parameter	−0.02(2)	−0.02(2)
Empirical formula	RbBa_3_(BSe_3_)_2_I	CsBa_3_(BSe_3_)_2_I
Formula weight	1119.77	1167.21
Temperature	173.0 K	273.15 K
Crystal system, space group	Orthorhombic, *Cmc*2_1_	Orthorhombic, *Cmc*2_1_
Unit cell dimensions	*a* = 16.3038(19) Å	*a* = 16.3293(17) Å
	*b* = 11.7474(11) Å	*b* = 11.8362(11) Å
	*c* = 8.5726(8) Å	*c* = 8.5707(6) Å
Volume	1641.9(3) Å^3^	1656.5(3) Å^3^
*Z*, calculated density	4, 4.530 g/cm^3^	4, 4.680 g/cm^3^
Flack parameter	−0.01(3)	0.04(3)

The SHG response is inherently dependent on the NCS crystal structure, and the analysis of NCS candidates is critical for the rational design of IR NLO materials. Thus, we conducted detailed analysis of the relationship between symmetry and the related features. By choosing the average deviation of space group number ($\hat{s}$) as an example, which is defined as:$\;\hat{s}=\sum_{i}x_{i}\left|s_{i}-\bar{s}\right|\;$, where *s_i_* means the space group number of element *i* in a compound, and *x_i_* means the atomic fraction. We calculated and analyzed the $\hat{s}$ values for all reported selenoborates, visualizing the distribution of $\hat{s}$ for NCS and CS structures using a box plot (Figure S9, Supporting Information). The results reveal a significant difference in the $\hat{s}$ distributions: the majority of NCS compounds exhibit $\hat{s}$ values above 90, while CS compounds predominantly fall within the range of 85–90. This suggests that a higher $\hat{s}$ value correlates with a greater likelihood of forming NCS selenoborates. That is to say, if the intrinsic symmetries of the constituted elements within a compound differ significantly, they may struggle to accommodate a highly symmetric arrangement when forming a stable compound, thereby favoring the adoption of a NCS structure. Statistical analysis regarding the influence of $\hat{s}$ on the formation of NCS structures provides a potentially valuable criterion for pre‐screening candidate formulas with a higher probability of yielding NCS strucutres, indicating its potential role to serve as a priori guidance for the future discovery of IR NLO materials.

### 2.5 Structural Characteristics of the ABa_3_(BSe_3_)_2_X Family

**Figure 3:**
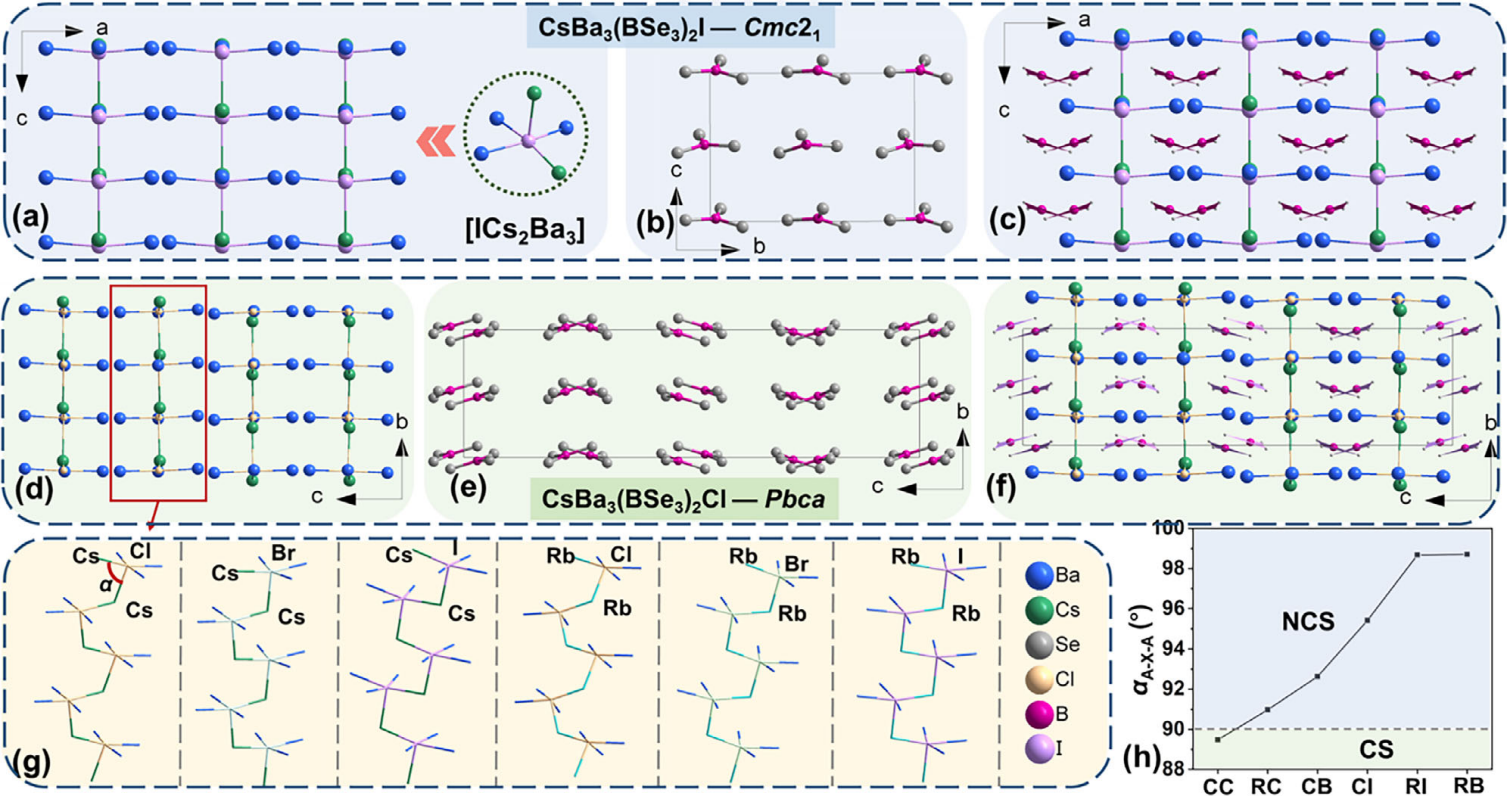
Crystal structure information of ABa_3_(BSe_3_)_2_X (A = Rb, Cs; X = Cl, Br, I). a) Structure of [ICs_2_Ba_3_] units and [CsBa_3_I] chains; b) [BSe_3_] anionic framework in CsBa_3_(BSe_3_)_2_I; c) The whole structure of CsBa_3_(BSe_3_)_2_I (NCS, *Cmc*2_1_); d) Structure of [CsBa_3_Cl] chains; e) [BSe_3_] anionic framework in CsBa_3_(BSe_3_)_2_Cl; f) The whole structure of CsBa_3_(BSe_3_)_2_Cl (CS, *Pbca*); g) structure of [A‐X‐A] chains in ABa_3_(BSe_3_)_2_X (A = Rb, Cs; X = Cl, Br, I); h) The relationship between symmetry and *α*_A‐X‐A_ (CC: CsBa_3_(BSe_3_)_2_Cl, RC: RbBa_3_(BSe_3_)_2_Cl, CB: CsBa_3_(BSe_3_)_2_Br, CI: CsBa_3_(BSe_3_)_2_I, RI: RbBa_3_(BSe_3_)_2_I, RB: RbBa_3_(BSe_3_)_2_Br).

To analyze their structures, we took CsBa_3_(BSe_3_)_2_I (NCS, *Cmc*2_1_) and CsBa_3_(BSe_3_)_2_Cl (CS, *Pbca*) as representative examples because of the existence of isostructures in this family. For the structures of CsBa_3_(BSe_3_)_2_I and CsBa_3_(BSe_3_)_2_Cl, they are both composed of isolated [BSe_3_] anion units and _∞_[CsBa_3_X]_n_ (X = Cl and I) electropositive chains. The notable structural distinction lies in the number of atoms in a unit cell, and the arrangement of [BSe_3_] units. In CsBa_3_(BSe_3_)_2_I (NCS, *Cmc*2_1_), an asymmetry unit comprises one Cs, two Ba, one B, three Se and one I atom. The I^−^ anion is coordinated with two Cs^+^ and three Ba^2+^ cations to form [ICs_2_Ba_3_] units. The [ICs_2_Ba_3_] units connect with each other by sharing Cs^+^ and Cl^−^ ions, forming electropositive _∞_[CsBa_3_X]_n_ (X = Cl, I) chains (**Figure** **3**a). While each B^3+^ is coordinated with three Se^2−^ ions to form [BSe_3_] anionic units with the B‐Se bond lengths ranging from 1.94 to 1.96 Å (Figure 3b). The overall structure could be envisioned as a combination of [CsBa_3_I] electropositive chains and anionic units [BSe_3_] (Figure 3c). Similarly, in CsBa_3_(BSe_3_)_2_Cl (CS, *Pbca*), the asymmetry unit includes one Cs, three Ba, two B, six Se, and one Cl atom. This compound comprises electropositive chains _∞_[CsBa_3_Cl]_n_ and anionic units [BSe_3_], with Cl^−^ and B^3+^ exhibiting identical coordination environments as in CsBa_3_(BSe_3_)_2_I (Figure 3d–f). Specially, the bond angle *α*_Cs‐I‐Cs_ (95.419 °) is larger than *α*_Cs‐Cl‐Cs_ (89.482 °), a disparity that may be attributed to the symmetry broken form CS CsBa_3_(BSe_3_)_2_Cl (*Pbca*) to NCS CsBa_3_(BSe_3_)_2_I (*Cmc*2_1_) (Figure 3g). Additionally, as depicted in Figure 3h, the angle *α*_A‐X‐A_ in the CS structure is smaller than 90 °, while NCS structures exhibit bond angles larger than 90°. In order to investigate whether the value of angel *α*_A‐X‐A_ is related to the structural symmetry, a survey was conducted on recently reported chalcohalides. As a result, this inference finds validation in various chalcohalides, such as [A_3_X][Ga_3_PS_8_] (A = K, Rb; X = Cl, Br), [ABa_2_Cl][Ga_4_S_8_] (A = Rb, Cs), Ba_3_AGa_5_Se_10_Cl_2_ (A = Cs, Rb, K) and so on.^[^ ^73^, ^91^, ^92^, ^93^ ^]^ This observation bears significant implications for the structural modulation from CS to NCS configurations.

### 2.6 Experimental Characterizations and DFT Analyses

**Figure 4:**
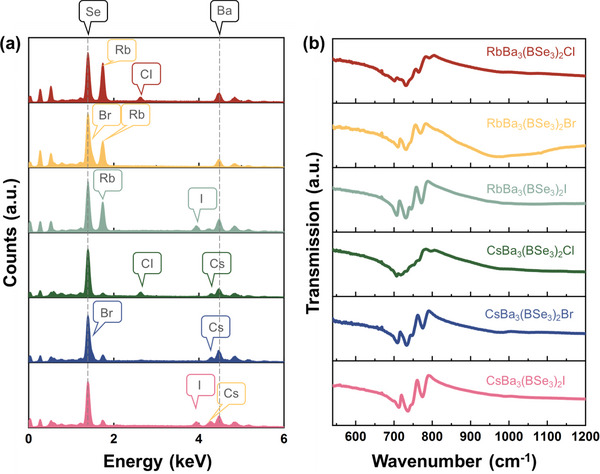
Experimental results of ABa_3_(BSe_3_)_2_X (A = Rb, Cs; X = Cl, Br, I). a) Energy dispersive X‐ray spectroscopy; b) IR absorption spectrum.

For the title compounds, we conducted multiple experimental characterizations, including powder X‐ray diffraction (PXRD), energy‐dispersive X‐ray spectroscopy (EDS), IR absorption spectrum to help verify the refined structures (**Figure** **4**; Figure S10, Supporting Information). Remarkably, the experimental XRD patterns match well with the calculated ones determined by the CIF files, affirming the successful synthesis of title compounds. Furthermore, the EDS results validate the existence of A, Ba, Se, X (A = Rb, Cs, X = Cl, Br, I) elements in title compounds (Figure 4a). The IR absorption spectra provide additional evidence for the presence of [BSe_3_] units (Figure 4b). By comparing the peaks intensity and frequency of groups within similar *D*_3__h_ species, the notable absorption peaks in the IR spectra, ≈700–800 cm^−1^, are attributed to the *v*_as_ vibrations for the pairs [BSe_3_]^3−^‐[BSe_3_].^3−[^ ^94^, ^95^ ^]^

**Figure 5:**
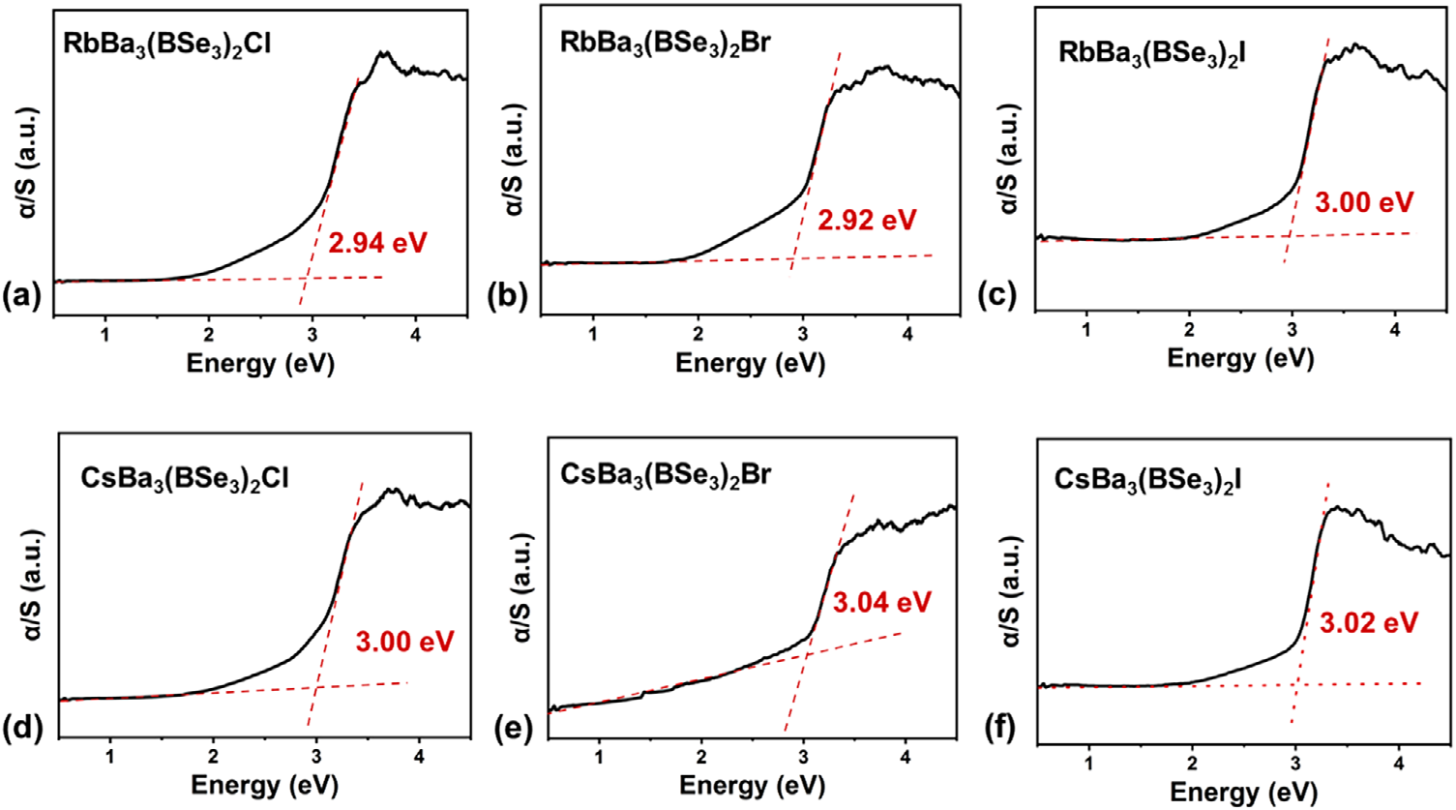
Experimental bandgaps of ABa_3_(BSe_3_)_2_X (A = Rb, Cs; X = Cl, Br, I). a) RbBa_3_(BSe_3_)_2_Cl; b) RbBa_3_(BSe_3_)_2_Br; c) RbBa_3_(BSe_3_)_2_I; d) CsBa_3_(BSe_3_)_2_Cl; e) CsBa_3_(BSe_3_)_2_Br; f) CsBa_3_(BSe_3_)_2_I.

**Figure 6:**
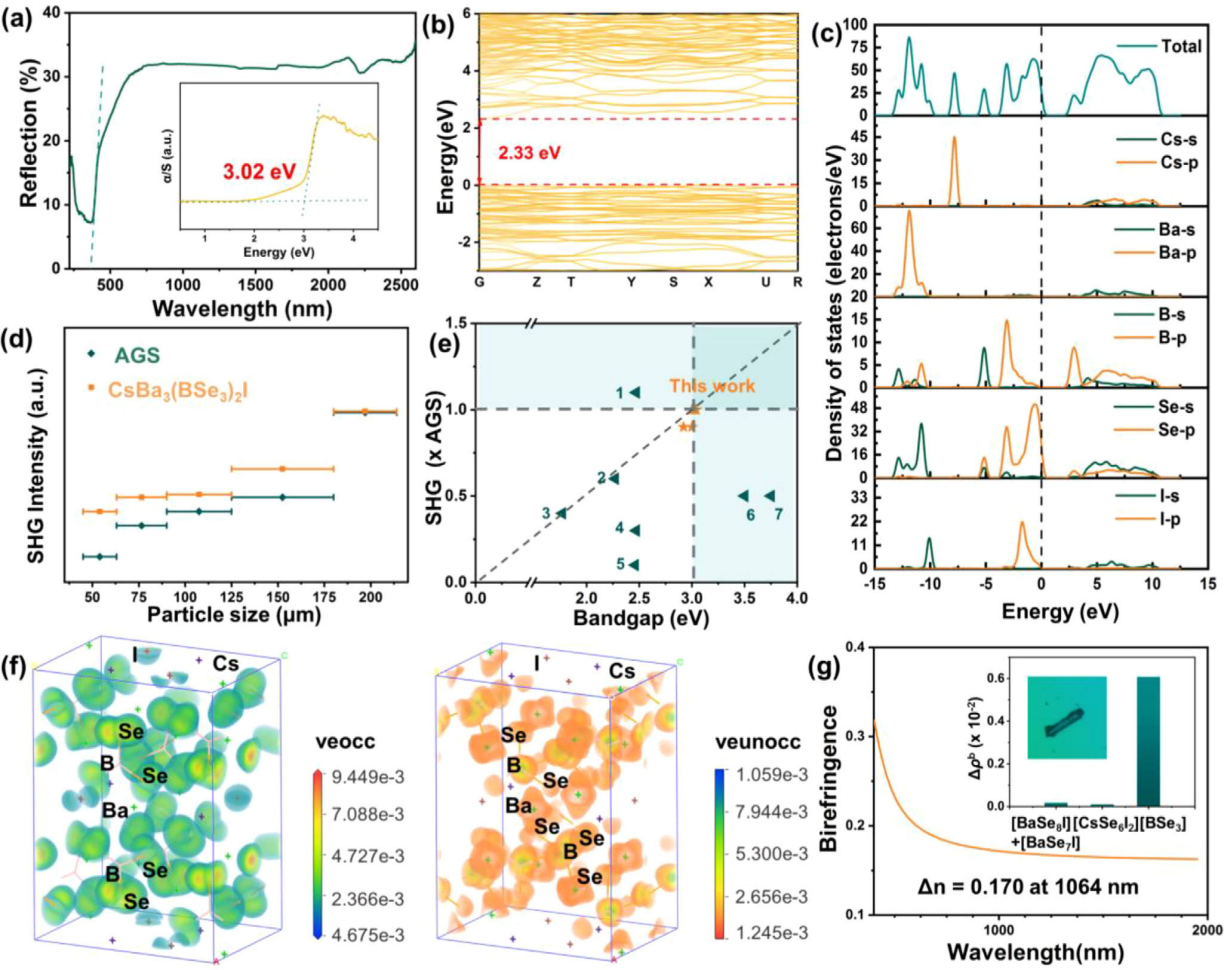
Experimental and calculated results of CsBa_3_(BSe_3_)_2_I. a) UV–vis‐NIR diffuse reflection spectrum; b) Band structures of CsBa_3_(BSe_3_)_2_l (GGA); c) Density of states of CsBa_3_(BSe_3_)_2_l; d) Experimental SHG intensity at 2.09 µm radiations (AGS used as the reference); e) Statistical analyses on the optical properties of several chalcogenides; f) SHG density maps of the occupied (left) and unoccupied (right) states in the VE process; g) Calculated birefringence curves, experimental birefringence measurement and the bonding electron density difference (Δ*ρ* ^b^) of different groups.

To investigate the bandgaps of ABa_3_(BSe_3_)_2_X (A = Rb, Cs; X = Cl, Br, I), the UV−vis−NIR diffuse reflection spectroscopy measurement, electronic structure calculations were applied. The bandgaps of ABa_3_(BSe_3_)_2_X (A = Rb, Cs; X = Cl, Br, I) were estimated by converting the reflectance spectra to absorbance using Kubelka‐Munk function. And their bandgaps range from 2.92 to 3.04 eV (**Figure** **5**). This series of compounds exhibit a comparatively large bandgap among chalcogenides, surpassing that of many renowned IR NLO materials, such as AGS (2.70 eV), *α*‐BaGa_4_Se_7_ (2.64 eV), *β*‐BaGa_4_Se_7_ (2.82 eV) and so on. Electronic band structures were further computed via DFT calculations. It was found that ABa_3_(BSe_3_)_2_X (A = Rb, Cs; X = Cl, Br, I) exhibit bandgaps ranging from 2.23 to 2.33 eV under the framework of PBE functional (Figure S11, Supporting Information), which is smaller than the experimental values mainly because of the inaccurately exchange correlation energy.^[^ ^96^, ^97^ ^]^ The electronic structure calculations based on Heyd‐Scuseria‐Ernzerhof (HSE06) hybrid functional was adopted, and the results show that ABa_3_(BSe_3_)_2_X (A = Rb, Cs; X = Cl, Br, I) exhibit bandgaps ranging from 2.99 to 3.08 eV, which are in good agreement with experimental values (Table S27, Supporting Information).^[^ ^98^ ^]^ Density of states (DOS) analysis reveals that Se‐3p and B‐2p orbitals predominantly give major contribution to the bandgap (Figure S12, Supporting Information). Taking CsBa_3_(BSe_3_)_2_I as an example for detailed analysis, it exhibits a direct bandgap of 3.02 eV (**Figure** **6**a,b). Near the Fermi level, Se‐3p orbitals dominate at the valence band maximum, while B‐2p orbitals predominate at the conduction band minimum, thus Se‐3p and B‐2p orbitals play a major role in defining the bandgap (Figure 6c).

The high LIDT of NLO crystals is paramount for practical applications, particularly when subjected to high‐power and high energy laser irradiation. Generally, LIDT is positively correlated with the bandgap. Accordingly, powder LIDTs of title compounds were evaluated through single‐pulse measurements. Powder samples were filled into the sample tray within a radiopaque enclosure, with AGS employed as the reference material. A pulsed laser beam (2090 nm, 50 ns, 3 Hz) was directed onto the sample surface, with the pulse energy progressively increased. An attenuator filter (80%) was utilized to mitigate the light energy impinging on the AGS sample. Visible damage under the microscope was observed for ABa_3_(BSe_3_)_2_X (A = Rb, Cs; X = Cl, Br, I) at laser energies ranging from 32.4 to 41.8 mJ (before attenuator filter), while AGS exhibits the damage at 7.8 mJ (before attenuator filter) (Table S28, Supporting Information). The results show that CsBa_3_(BSe_3_)_2_I has a high LIDT of ≈ 5 × AGS, which are higher than that of Na_2_Hg_3_Ge_2_S_8_ (3× AGS), Na_2_Hg_3_Sn_2_S_8_ (1× AGS), Na_2_ZnSn_2_S_6_ (2× AGS) etc. (Table S29, Supporting Information).

To investigate the SHG responses of title compounds, the Kurtz and Perry method and SHG density method were employed (Figure S13, Supporting Information). The result reveals that the five NCS compounds in ABa_3_(BSe_3_)_2_X (A = Rb, Cs; X = Cl, Br, I) exhibit large SHG response of 0.9‐1.0× AGS. Taking CsBa_3_(BSe_3_)_2_I as an example for detailed analysis, it exhibits a substantial SHG response of ≈ 1.0 times that of AGS with phase matching behavior (Figure 6d), which is related to the high hyperpolarizability of [BSe_3_]. For IR NLO material, their bandgaps and SHG are mutually restrictive. Numerous compounds with wide bandgaps exhibit small SHG responses, such as RbCd_4_Ga_3_S_9_ (3.73 eV, 0.01× AGS), Na_2_Ga_2_SiS_6_ (3.93 eV, 0.2× AGS). Conversely, many compounds with large SHG responses possess narrow bandgaps, like BaGa_2_SnSe_6_ (5.2× AGS, 1.95 eV), RbInSn_2_Se_6_ (4.8× AGS, 1.8 eV) and RbGaSn_2_Se_6_ (4.2× AGS, 1.92 eV). CsBa_3_(BSe_3_)_2_I exhibits good balance between a wide bandgap and a large SHG response (Figure 6e and Table S30, Supporting Information). The SHG density maps of the unoccupied and occupied states in the virtual electron process suggest that the SHG mainly originates from the [BSe_3_] units (Figure 6f).

**Figure 7:**
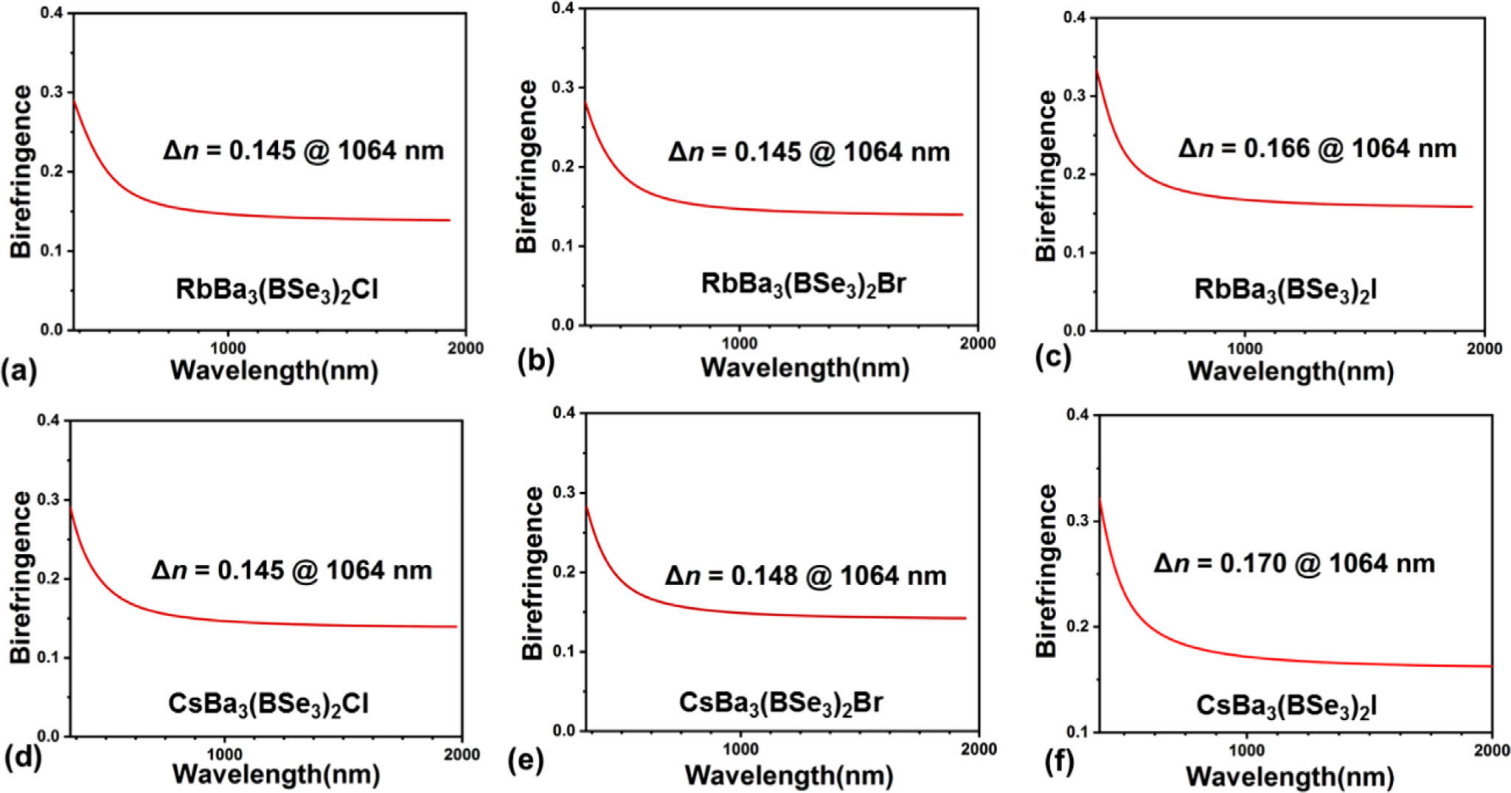
Calculated birefringence of ABa_3_(BSe_3_)_2_X (A = Rb, Cs; X = Cl, Br, I). a) RbBa_3_(BSe_3_)_2_Cl; b) RbBa_3_(BSe_3_)_2_Br; c) RbBa_3_(BSe_3_)_2_I; d) CsBa_3_(BSe_3_)_2_Cl; e) CsBa_3_(BSe_3_)_2_Br; f) CsBa_3_(BSe_3_)_2_I.

The birefringence is also important to IRFMs, therefore relevant experimental and theoretical researches were conducted on it. The calculated birefringence of ABa_3_(BSe_3_)_2_X (A = Rb, Cs; X = Cl, Br, I) is ≈ 0.145 – 0.170 at 1064 nm (**Figure** **7**). Taking CsBa_3_(BSe_3_)_2_I as an example for detailed analysis, the experimental refractive index of CsBa_3_(BSe_3_)_2_I was evaluated through a Carl Zeiss Axioscope 5 polarizing microscope.^[^ ^99^ ^]^ The retardation (*R_e_*) value was determined to be 1.17 µm and the thickness (*T*) of flat crystal selected is 11 µm. According to the formula *R_e_* = |*N*_2_ – *N*_1_| × *T* = Δ*n* × *T*, the experimental refractive index difference of CsBa_3_(BSe_3_)_2_I at 546 nm was determined to be 0.106, which proves CsBa_3_(BSe_3_)_2_I exhibits large birefringence experimentally. What's more, the response electron distribution anisotropy (REDA)^[^ ^100^ ^]^ analysis was adopted to investigate the origin of the birefringence. The analysis demonstrates that the significant birefringence mainly originates from the π‐conjugated [BSe_3_] (Figure 6g).

## 3 Conclusion

In conclusion, we developed a synergistic framework based on interpretable ML, which accelerates the discovery of a series of novel high‐performance IRFMs. An IRFM predictor based on two ML classifiers for predicting SF and *E*_g_ with great accuracy is trained, in which some prior domain knowledge for chalcogenides based on model interpretability is inferred. The pre‐trained IRFM predictor effectively guide us in synthesizing the first reported selenoborate halides, ABa_3_(BSe_3_)_2_X (A = Rb, Cs; X = Cl, Br, I), which exhibit well balanced property with wide bandgaps (2.92‐3.04 eV), large SHG (0.9‐1.0× AGS), moderate birefringence (0.145 – 0.170 at 1064 nm) and high LIDTs (4‐6× AGS), illustrating their potential as high‐performance IRFMs. The structure‐property analyses based on first principles theory demonstrate that [BSe_3_] is the origin of optical properties, indicating that [BSe_3_] is a good gene to obtain high performed IRFMs. Ultimately, we established an IRFM design strategy characterized by ML‐based efficient screening followed by precise experimental validation. This work promotes the cooperation of ML technology and materials science, introducing a novel research system focused on selenoborates.

## 4 Experimental Section

### Microscopic Unit Calculations

The Gaussian 09 package was employed to explore the linear and nonlinear properties of related anionic groups, including [BO_3_], [BO_4_], [BS_3_], [BS_4_], [BSe_3_], [BSe_4_], [GaS_4_] and [GaSe_4_] groups. B3LYP (Becke, three‐parameter, Lee‐Yang‐Parr) exchange‐correlation functional with the Lee‐Yang‐Parr correlation functional at the 6–31G basis set was adopted.

### Machine Learning Models

To ensure the robustness of this approach, nine widely used classification algorithms were adopted based on Scikit‐learn package,^[^ ^82^ ^]^ which contain Support Vector Machines (M1),^[^ ^101^ ^]^ Decision Tree Classifier (M2),^[^ ^102^ ^]^ AdaBoost Classifier (M3),^[^ ^103^ ^]^ Gradient Boosting Classifier (M4),^[^ ^104^ ^]^ Random Forest Classifier (M5),^[^ ^105^ ^]^ XGBoost Classifier (M6),^[^ ^106^ ^]^ Extra Trees Classifier (M7),^[^ ^107^ ^]^ LightGBM Classifier (M8)^[^ ^108^ ^]^ and CatBoost Classifier (M9).^[^ ^109^, ^110^ ^]^ Each algorithm has its own strengths and limitations, so it was quantified their performance on this dataset to select the most suitable model. Taking the training process for predicting SF as an example, it was assessed model performance using the Area under the Receiver Operating Characteristic (ROC) Curve (AUC) and accuracy (Figure S4, Supporting Information). Models like Random Forest and Extra Trees showed good performance but were outperformed by LightGBM in terms of ROC AUC and accuracy. CatBoost achieved the highest accuracy, but its lower ROC AUC suggested potential overfitting. LightGBM balanced both metrics, making it the optimal choice for the SF classification task. For *E*_g_ prediction, it was selected XGBoost after the similar comparative analysis, where it demonstrated excellent performance and robustness against overfitting.

### First‐Principles Calculations

The first‐principles calculations were implemented in the CASTEP using a planewave pseudopotential method based on density function theory (DFT) to obtain the electronic structures and optical properties for ABa_3_(BSe_3_)_2_X (A = Rb, Cs; X = Cl, Br, I).^[^ ^111^ ^]^ The exchange correlation functional and pseudopotential for the title compounds were generalized gradient approximation (GGA) with Perdew‐Burke‐Ernzerhof (PBE) functional with the energy cutoff of 540 eV, respectively.^[^ ^96^, ^112^ ^]^ The *k*‐point separation for RbBa_3_(BSe_3_)_2_X (X = Cl, Br, I) and CsBa_3_(BSe_3_)_2_X (X = Cl, Br, I) were set as 3×3×5, 2×3×5, 2×3×5, 3×3×1, 3×4×6, 2×3×5 in the Brillouin zone, respectively. Norm‐conserving pseudopotentials (NCP) were employed for each atomic species with the following valence configurations: A‐4*s* ^2^4*p* ^6^5*s* ^1^, Ba‐4*s* ^2^4*p* ^6^5*s* ^2^, B‐2*s* ^2^2*p* ^1^, Se‐3*s* ^2^3*p* ^4^, X‐4*s* ^2^4*p* ^5^ (A = Rb, Cs; X = Cl, Br, I).^[^ ^113^ ^]^ Other calculation parameters and convergent criteria were set as the default values of the CASTEP package.^[^ ^114^ ^]^ The bandgap difference between the experimental values and the values calculated by GGA method was used as the scissors operation to calculate the birefringence and the SHG coefficients. The corresponding values of scissors operation were set as 0.69, 0.69, 0.72, 0.74, 0.77 and 0.69 eV. In addition, the Heyd‐Scuseria‐Ernzerhof (HSE06) hybrid functional was adopted for more accurate bandgap values.

### Calculation Methods for Optical Properties

The calculations of the linear optical performance were described in terms of the complex dielectric constant *ε*(*ω*) = *ε*_1_(*ω*) + i*ε*_2_(*ω*). The imaginary part *ε*_2_(*ω*) of the dielectric function *ε*(*ω*) was calculated by using momentum matrix elements between the occupied and unoccupied electronic states:

(1) $$\varepsilon_{2}\left(\hbar \omega \right)=\frac{2e^{2}\pi }{\Omega \varepsilon_{0}}\sum_{kcv}{\left|\left\langle \psi_{k}^{c}\left|\hat{u}\cdot r\right|\psi_{k}^{v}\right\rangle \right|}^{2}\delta \left[E_{k}^{c}-E_{k}^{v}-E\right]$$

Here *Ω* is the unit cell volume, ν and *c* represent the valence bands and conduction bands, respectively. ω and $\hat{u}$ are the frequency and the unit vector in the polarization direction of the incident light. Under the periodic boundary condition, $|\left\langle \psi_{k}^{c}|\hat{u}\cdot r|\psi_{k}^{v}\right\rangle |$ is the transition matrix element between the valence bands and the conduction bands at a specific *k* point in the first Brillouin zone. The real part *ε*_1_(*ω*) can be obtained from the imaginary part *ε*_2_(*ω*) by using the Kramers‐Kronig transformation. The refractive index *n* and birefringence ∆*n* can be obtained from the complex dielectric function.^[^ ^115^ ^]^

Furthermore, the response electron distribution anisotropy (REDA) method was employed to identify the contribution of each group to birefringence and the birefringence can be estimated as follow:^[^ ^100^ ^]^

(2) $$\Delta n=\frac{ℜ\sum_{g}{\left[N_{c}Z_{a}\Delta \rho^{b}\right]}_{g}}{2n_{1}E_{o}}$$

Here, ℜ is the correction coefficient, *N*_c_ is the coordination number of the nearest neighbor cations to the central anion, *Z*_a_ is the formal chemical valence of the anion, Δ*ρ* ^b^ is the difference between the maximum and minimum of the covalent electron density of the covalent bond on the optical principal axes of a crystal, *E*_o_ is the optical bandgap, *n*_1_ is the minimum refractive index.

*Synthesis of ABa_3_(BSe_3_)_2_X (A = Rb, Cs; X = Cl, Br, I)*: The crystals of RbBa_3_(BSe_3_)_2_X (X = Cl, Br, I) for single crystal X‐ray diffraction were obtained by spontaneous crystallization method. The synthesis processes are as follows: 1) A mixture of RbX (X = Cl, Br, I), BaSe, B and Se with mole ratio of 1:3:2:3 was mixed in an agate mortar, which was placed in the graphite crucible. 2) The crucible was covered with a tight crucible cap and then moved into a glassy carbon‐inwall coated silica tube. 3) The crucible was sealed by oxyhydrogen flame under a vacuumed environment (10^−4^ Pa). 4) After that, the sealed tube was put into a temperature program‐controlled furnace with the heating progress as follow: 30 °C (40 °C/h) → 700 °C (keep for 50 h) (3 °C/h) → 300 °C (60 °C/h) → 30 °C. The crystals of CsBa_3_(BSe_3_)_2_X (X = Cl, Br, I) for single crystal X‐ray diffraction were obtained by spontaneous crystallization method. The synthesis process is similar to that of RbBa_3_(BSe_3_)_2_X (X = Cl, Br, I) except the temperature progress is as follows: 30 °C (40 °C/h) →300 °C (keep for 5 h) (45 °C/h) → 730 °C (keep for 50 h) (3 °C/h) → 400 °C (57 °C/h) → 30 °C.

### Single X‐Ray Diffraction Measurement and Structure Refinement

The structure data of ABa_3_(BSe_3_)_2_X (A = Rb, Cs; X = Cl, Br, I) were collected on a Bruker SMART APEX II 4K CCD diffractometer equipped with Mo K*α* radiation (λ = 0.71073 Å) operating at 50 kV and 40 mA at room temperature. The data were refined through full‐matrix least‐squares on *F* ^2^ using SHELXTL program package. Structure determination was based on the direct method, and the face‐indexed absorption correction was made with XPREP Program. The final structure was checked with PLATON and no higher symmetries were found. During the analysis process, it was found that the Flack was too large and the *R* value was too high and Platon detected inverted twins, the matrix was (‐1,0,0,0,1,0,0,0,‐1). By adding twin instructions, the *R* value and Flack were decreased to a reasonable level.

### Powder X‐Ray Diffraction Measurement

X−ray diffraction (XRD) characterization for ABa_3_(BSe_3_)_2_X (A = Rb, Cs; X = Cl, Br, I) was implemented on an automated Bruker D2 X‐ray diffractometer from 10 to 70 ° (2θ) with a scan step width of 0.02 ° and a fixed counting time of 1 s/step.

### Energy Dispersive X‐Ray Spectroscopy

Elemental analysis was carried on clean single crystal surfaces with the aid of a field emission scanning electron microscope (SEM, SUPRA 55VP) equipped with an energy dispersive X‐ray spectroscope (EDX, BRUKER x‐flash‐sdd‐5010).

### Infrared Absorption Spectrum

The IR spectrum was recorded on a Shimadzu IR Affinity‐1 Fourier transform infrared spectrometer with a resolution of 2 cm^−1^, covering the wavenumber range of 400–4000 cm^−1^. The crystal samples and KBr were mixed in the ratio of ≈ 1:100, dried, and ground into fine powder and then pressed into a transparent sheet on the tablet machine. The sheet was loaded in the sample chamber, and then the IR absorption spectrum was measured.

### UV–vis–NIR Diffuse Reflectance Spectroscopy

Optical diffuse reflectance spectra of ABa_3_(BSe_3_)_2_X (A = Rb, Cs; X = Cl, Br, I) were measured at 298 K on Shimadzu SolidSpec‐3700DUV spectrophotometer with a wavelength range of 180–2600 nm, which can provide the visible or UV cut‐off edge. The experimental bandgaps of ABa_3_(BSe_3_)_2_X (A = Rb, Cs; X = Cl, Br, I) can be estimated by converting the reflectance spectra to absorbance using Kubelka‐Munk function.^[^ ^116^ ^]^

### Birefringence Measurement

The birefringence of CsBa_3_(BSe_3_)_2_I was characterized by using the polarizing microscope equipped (ZEISS Axio Scope. A1) with Berek compensator. The wavelength of the light source was 546 nm. The difference in the optical path (*R*) for one direction was determined according to the interference color with the maximum value of the crystal under the polarized light. The formula for calculating the birefringence was listed below,

*R* = |*N*_2_ – *N*_1_| × *T* = Δ*n* × *T*. Herein, Δ*n* means the difference of refractive index, and *T* denotes the thickness of the crystal.

### Powder Laser‐Induced Damage Threshold Measurement

On the same measurement condition, the powder LIDTs of ABa_3_(BSe_3_)_2_X (A = Rb, Cs; X = Cl, Br, I) were evaluated by a single‐pulse measurement method, with AgGaS_2_ as the reference. The samples of ABa_3_(BSe_3_)_2_X (A = Rb, Cs; X = Cl, Br, I) and AgGaS_2_ were finely ground and sieved, among which samples with a particle size among 45–63 *µm* were collected and pressed into the sample trays. The samples were exposed to a pulsed laser beam (2.09 *µm*, 50 ns, 3 Hz) while the pulse energy was continuously increased. Energy increase was stopped when a damage occurrence (color change) was detected on the sample surface under a microscope. The LIDTs was calculated using the formula: $LIDT=\frac{E}{s\times \tau }\;$, where *E* is the laser energy, *s* is the beam spot area, and *τ* is the pulse widths.

### Second‐Harmonic Generation Measurement

The SHG responses of ABa_3_(BSe_3_)_2_X (A = Rb, Cs; X = Cl, Br, I) were measured through the Kurtz−Perry method^[^ ^117^ ^]^ with a 2.09 *µm* Q‐switch laser. The ABa_3_(BSe_3_)_2_X (A = Rb, Cs; X = Cl, Br, I) samples were grounded and mixed. The CsBa_3_(BSe_3_)_2_I samples were ground and sieved into different particle sizes: 45−63, 63−90, 90−125, 125−180, and 180−212 *µm*. These samples were placed between two glass microscope slides and fixed with a 1 mm‐thick, 8 mm‐diameter silicone. Then, they were placed into a Q‐switched Ho:Tm:Cr:YAG laser with a wavelength of 2.09 *µm*, and the SHG signals were recorded by an oscilloscope connected to the laser. The AgGaS_2_ samples with the same particle sizes were used as the references.

[CCDC 2351981–2351986 contains the supplementary crystallographic data for this paper. These data can be obtained free of charge from The Cambridge Crystallographic Data Centre via www.ccdc.cam.ac.uk/data_request/cif.]

## Conflict of Interest

The authors declare no conflict of interest.

## Supporting information



Supporting Information

## Data Availability

The data that support the findings of this study are available in the supplementary material of this article.
