# Supplementary material for: Synergistic Machine Learning Guided Discovery of ABa3(BSe3)2X (A = Rb, Cs; X = Cl, Br, I): A Promising Family as Property‐Balanced IR Functional Materials
Source: Adv Sci (Weinh). 2025 Apr 26;12(23):2417851. doi: 10.1002/advs.202417851 (PMC12199391; doi:10.1002/advs.202417851)
Supplement: Supplementary file 1 — Supporting Information [file ADVS-12-2417851-s001.pdf]

ADVANCED SCIENCE
Open Access

Supporting Information

for *Adv. Sci.*, DOI 10.1002/advs.202417851

Synergistic Machine Learning Guided Discovery of $ABa_3(BSe_3)_2X$ (A = Rb, Cs; X = Cl, Br, I): A Promising Family as Property-Balanced IR Functional Materials

Yihan Yun, Mengfan Wu, Zhihua Yang, Guangmao Li and Shilie Pan**

Supporting Information

Synergistic Machine Learning Guided Discovery of $ABa_3(BSe_3)_2X$ (A = Rb, Cs; X = Cl, Br, I): A Promising Family as Property-Balanced IR Functional Materials

Yihan Yun, [#] *Mengfan Wu,* [#] *Zhihua Yang, Guangmao Li,* [*] *and Shilie Pan* [*]

Y. Yun, M. Wu, Z. Yang, G. Li, S. Pan

Research Center for Crystal Materials; State Key Laboratory of Functional Materials and Devices for Special Environmental Conditions; Xinjiang Key Laboratory of Functional Crystal Materials; Xinjiang Technical Institute of Physics and Chemistry, Chinese Academy of Sciences, 40-1 South Beijing Road, Urumqi 830011, China.

E-mails: slpan@ms.xjb.ac.cn; ligm@ms.xjb.ac.cn

Y. Yun, Z. Yang, G. Li, S. Pan

Center of Materials Science and Optoelectronics Engineering, University of Chinese Academy of Sciences, Beijing 100049, China.

Table S1. Selected structure information of reported selenoborates.

Formula	Space group	Lattice parameters	Ref.
Tl_3BSe_3	$P2_1/m$ (No. 11)	a = 5.547(2) b = 10.099(3) c = 6.852(2) β =97.59(3)	[1]
$Ba_7(BSe_3)_4Se$	C2/c (No. 15)	a = 10.513(2) b = 25.021(5) c = 10.513(2) β = 90.10(3)	[2]
$Ba_3(BSe_3)(SbSe_3)$*	$P\bar{6}2m$ (No. 189)	a = 17.720(4) c = 11.251(3)	[3]
$Li_{6-2x}Sr_{2+x}B_{10}Se_{20}$ (x ≈ 0.7)	$I4_1/a$ (No. 88)	a = 14.735(6) c = 14.145(12)	[4]
$Na_2B_2Se_7$	$C2/c$ (No. 15)	a = 11.863(4) b = 6.703(2) c = 13.811(2) β = 109.41(2)	[5]
$K_2B_2Se_7$	$C2/c$ (No. 15)	a = 12.092(4) b = 7.054(2) c = 13.991(5) β = 107.79(3)	[5]
$CsBSe_3$	$P2_1/c$ (No. 14)	a = 7.570(2) b = 12.791(4) c = 6.171(2) β = 107.09(2)	[6]
$RbBSe_3$	$P2_1/c$ (No. 14)	a = 7.2789(15) b =12.385(3) c = 6.1690(12) β = 105.67(3)	[6]
$TlBSe_3$*	Cc (No. 9)	a = 6.1662(12) b = 12.109(2) c = 7.0311(14) β = 113.88(3)	[6]
$Cs_3B_3Se_{10}$	$P\bar{1}$ (N0. 2)	a = 7.5831(15) b = 8.4643(17) c = 15.276(3) a = 107.03(3) β = 89.29(3) γ = 101.19(3)	[7]
$Tl_3B_3Se_{10}$	$P\bar{1}$ (N0. 2)	a = 7.0989(14) b = 8.0724(16) c = 14.545(3) α = 105.24(3) β = 95.82(3) γ = 92.79(3)	[7]
$Tl_2B_2Se_7$	$C2/c$ (No. 15)	a = 11.878(2) b = 7.0909(14) c = 13.998(3) β =108.37(3)	[7]
$Rb_2B_2Se_7$	$C2/c$ (No. 15)	a = 12.414(3) b = 7.314(2) c = 14.092(3) β = 90107.30(3)	[7]
$Ba_2B_4Se_{13}$	$P2_1/c$ (No. 14)	a = 12.790(3) b = 11.560(2) c = 12.862(3) β = 103.22(3)	[8]
BaB_2Se_6	$Cmca$ (No. 64)	a = 11.326(2) b = 7.6587(15) c = 10.315(2)	[8]
$Li_{6-x}Cs_xB_{10}Se_{18}$ (x ≈ 1)	$I4_1/amd$ (No. 141)	a = 10.0285(14) b = 10.0285(14) c = 27.743(6)	[9]
$CuBSe_2$*	$I\bar{4}2d$ (No. 122)	a = 5.539 b = 5.539 c = 10.734	[10]
$Li_2B_2Se_5$	$C2/c$ (No. 15)	a = 10.616(2) b = 5.3181(11) c = 12.382(3) β = 109.96(3)	[11]
$Na_6B_{10}Se_{18}$	$I41/acd$ (No. 142)	a = 15.128(2) b = 15.128(2) c = 27.955(6)	[12]
$Li_{6+2x}[B_{10}Se_{18}]Se_x$ (x ≈ 2)	$C2/c$ (No. 15)	a = 17.411(1) b = 21.900(1) c = 17.820(1) β = 101.6(1)	[13]
$Li_7B_7Se_{15}$	$P42/nbc$ (No. 133)	a = 11.4107(4) c = = 16.4251(5)	[14]
$RbBa_3(BSe_3)_2Cl$*	$Cmc2_1$ (No. 36)	a = 15.403(2) b = 11.8262(18) c = 8.6681(14)	a
$RbBa_3(BSe_3)_2Br$*	$Cmc2_1$ (No. 36)	a = 16.0147(11) b = 11.6688(7) c = 8.5898(5)	a
$RbBa_3(BSe_3)_2I$*	$Cmc2_1$ (No. 36)	a = 16.3038(19) b = 11.7474(11) c = 8.5726(8)	a
$CsBa_3(BSe_3)_2Cl$	$Pbca$ (No. 61)	a = 11.8928(8) b = 8.6874(5) c = 31.030(2)	a
$CsBa_3(BSe_3)_2Br$*	$Cmc2_1$ (No. 36)	a = 16.1046(13) b =11.7634(10) c =8.5819(6)	a
$CsBa_3(BSe_3)_2I$*	$Cmc2_1$ (No. 36)	a = 16.3293(17) b =11.8362(11) c =8.5707(6)	a

*: Non-centrosymmetric compound

a: This work

Table S2. 55 feature descriptors used in the LightGBM model for predicting SF.

Lable	Abbreviation	Feature descriptors	Descriptions
F1	/	MagpieData mean NUnfilled	Mean number of unfilled valence orbitals
F2	/	MagpieData avg_dev NUnfilled	Average deviation of number of unfilled p orbitals
F3	/	MagpieData mean NpUnfilled	Mean number of unfilled p orbitals
F4	/	MagpieData mean Column	Mean periodic table column
F5	$\hat{s}$	MagpieData avg_dev SpaceGroupNumber	Average deviation of space number
F6	/	MagpieData mean Electronegativity	Mean electronegativity
F7	/	MagpieData avg_dev Nvalence	Average deviation of number of valence electrons
F8	/	MagpieData avg_dev NpUnfilled	Average deviation of number of unfilled p orbitals
F9	$\overline{\mathrm{V}}$	MagpieData mean GSvolume_pa	Mean DFT-computed volume of elemental solid
F10	L_2	2-norm	2-norm of stoichiometric attributes
F11	/	MagpieData avg_dev GSvolume_pa	Average deviation of DFT-computed volume of elemental solid
F12	/	MagpieData avg_dev MeltingT	Average deviation of melting temperature
F13	$\hat{\mathrm{E}}_{\mathrm{g}}^{\mathrm{GS}}$	MagpieData avg_dev GSbandgap	Average deviation of DFT band gap of elemental solid
F14	$\overline{\mathrm{V}}_{\mathrm{SG}}$	MagpieData mean SpaceGroupNumber	Average deviation of space group number
F15	/	MagpieData avg_dev Electronegativity	Average deviation of electronegativity
F16	/	MagpieData mean NValence	Mean number of valence electrons
F17	/	MagpieData mean MeltingT	Mean melting temperature
F18	/	MagpieData mean CovalentRadius	Mean covalent radius
F19	/	MagpieData avg_dev NpValence	Average deviation of number of filled p orbitals
F20	/	MagpieData avg_dev CovalentRadius	Average deviation of covalent radius
F21	P	Boolean value compound possible	a Boolean denoting whether it is possible to form a neutral, ionic compound

F22	/	MagpieData range GSvolume_pa	Range DFT-computed volume of elemental solid
F23	/	MagpieData avg_dev NdValence	Average deviation of number of filled d orbitals
F24	/	MagpieData avg_dev NdUnfilled	Average deviation of number of unfilled d orbitals
F25	/	MagpieData avg_dev Row	Average deviation of periodic table row among elements in composition
F26	V^{min}	MagpieData minimum GSvolume_pa	Minimum DFT-computed volume of elemental solid
F27	/	LUMO_energy	Lowest unoccupied molecular orbital energy
F28	/	HOMO_energy	Highest occupied molecular orbital energy
F29	χ^{min}	MagpieData minimum Electronegativity	Minimum electronegativity
F30	/	MagpieData mean Row	Mean periodic table row among elements in composition
F31	/	MagpieData avg_dev MendeleevNumber	Average deviation of Mendeleev number
F32	/	MagpieData mean GSbandgap	Mean DFT band gap of elemental solid
F33	/	MagpieData range Electronegativity	Range electronegativity
F34	/	MagpieData range MeltingT	Range melting temperature
F35	N^{min}	MagpieData minimum Number	Minimum atomic number
F36	/	MagpieData range NUnfilled	Range number of unfilled valence orbitals
F37	/	MagpieData minimum NUnfilled	Minimum number of unfilled valence orbitals
F38	/	MagpieData avg_dev NsValence	Average deviation of number of filled s orbitals
F39	E_g^{HL}	gap_AO	Estimated band gap from HOMO and LUMO energies
F40	/	MagpieData avg_dev AtomicWeight	Average deviation of atomic weight among elements
F41	$\hat{\mu}$	MagpieData avg_dev GSmagmom	Average deviation of magnetic moment of the elemental solids
F42	/	MagpieData mean NpValence	Mean number of filled p orbitals
F43	/	MagpieData mean NdValence	Mean number of filled d orbitals

F44	/	MagpieData range CovalentRadius	Range covalent radius
F45	/	MagpieData mode GSvolume_pa	Mode DFT-computed volume of elemental solid
F46	/	MagpieData range MendeleevNumber	Range Mendeleev number
F47	/	MagpieData range NValence	Range number of valence electrons
F48	/	MagpieData mean NsUnfilled	Mean number of unfilled s orbitals
F49	/	MagpieData minimum SpaceGroupNumber	Minimum space number
F50	/	MagpieData minimum MeltingT	Minimum melting temperature
F51	/	MagpieData mode MendeleevNumber	Mode mendeleev number
F52	/	MagpieData range SpaceGroupNumber	Range space number
F53	/	MagpieData maximum Number	Maximum atomic number
F54	/	0-norm	0-norm of stoichiometric attributes
F55	/	MagpieData range NpUnfilled	Range number of unfilled p orbitals

Table S3. The optimal hyperparameters of LightGBM model.

Hyperparameter	Value
lambda_l1	$7.82e^{-6}$
lambda_l2	0.002014482
num_leaves	64
feature_fraction	0.716616278
bagging_fraction	0.984832202
bagging_freq	5
min_child_samples	14
learning_rate	0.07318074

Table S4. 44 feature descriptors used in the XGBoost model for predicting E_g.

Lable	Abbreviation	Feature descriptors	Descriptions
F1	$\overline{N}_{d,V}$	MagpieData mean NdValence	Mean number of filled d orbitals
F2	/	MagpieData minimum CovalentRadius	Minimum covalent radius
F3	$\overline{N}_{d,U}$	MagpieData mean NdUnfilled	Mean number of unfilled d orbitals
F4	T_M^{max}	MagpieData maximum MeltingT	Maximum melting temperature
F5	/	MagpieData maximum SpaceGroupNumber	Maximum space number
F6	/	MagpieData maximum NpUnfilled	Maximum number of unfilled p orbitals
F7	/	MagpieData mode NpUnfilled	Mode number of unfilled p orbitals
F8	/	MagpieData range Row	Range periodic table row among elements in composition
F9	/	MagpieData maximum NdUnfilled	Maximum number of unfilled d orbital
F10	/	HOMO_energy	Highest occupied molecular orbital energy
F11	V^{min}	MagpieData minimum GSvolume_pa	Minimum DFT-computed volume of elemental solid
F12	/	MagpieData avg_dev NdValence	Average deviation of number of filled d orbital
F13		MagpieData mean GSmagmom	Mean magnetic moment of the elemental solids
F14		MagpieData maximum MendeleevNumber	Maximum mendeleev number
F15	/	MagpieData minimum NpUnfilled	Minimum number of unfilled p orbitals
F16	$\overline{N}_{s,V}$	MagpieData mean NsValence	Mean number of filled s orbitals
F17	/	MagpieData range NUnfilled	Range number of unfilled valence orbitals
F18	/	MagpieData maximum GSbandgap	Maximum DFT band gap of elemental solid
F19	/	MagpieData mode NUnfilled	Mode number of unfilled valence orbitals
F20	$\overline{N}_V$	MagpieData mean NValence	Mean number of valence electron
F21	/	MagpieData mean MeltingT	Mean melting temperature

F22	$\hat{\chi}$	MagpieData avg_dev Electronegativity	Average deviation of electronegativity
F23	/	MagpieData mode MeltingT	Mode melting temperature
F24	$\bar{R}$	MagpieData mean Row	Mean periodic table row among elements in composition
F25	/	MagpieData range Column	Range periodic table column
F26	/	Magpie Data mean NpValence	Mean number of filled p orbitals
F27	/	MagpieData minimum MeltingT	Minimum melting temperature
F28	/	MagpieData avg_dev MendeleevNumber	Average deviation of mendeleev number
F29	/	MagpieData mode NValence	Mode number of valence electron
F30	/	MagpieData range Electronegativity	Range electronegativity
F31	L_2	2-norm	2-norm of stoichiometric attributes
F32	/	MagpieData avg_dev NValence	Average deviation of number of valence electron
F33	/	MagpieData mean NUnfilled	Mean number of unfilled valence orbitals
F34	/	MagpieData minimum NsValence	Minimum number of filled s orbitals
F35	/	MagpieData minimum GSbandgap	Minimum DFT band gap of elemental solid
F36	/	MagpieData range NpUnfilled	Range number of unfilled p orbitals
F37	/	MagpieData mean NpUnfilled	Mean number of unfilled p orbitals
F38	/	MagpieData mean Column	Mean periodic table column
F39	/	MagpieData avg_dev Number	Average deviation of atomic number
F40	/	LUMO_energy	Lowest unoccupied molecular orbital energy
F41	/	MagpieData minimum Electronegativity	Minimum electronegativity
F42	E_g^{HL}	gap_AO	Estimated band gap from HOMO and LUMO energies
F43	/	MagpieData avg_dev NpUnfilled	Average deviation of number of unfilled p orbitals

Table S5. The optimal hyperparameters of XGBoost model.

Hyperparameter	Value
lambda	$3.27e^{-5}$
alpha	0.49311197300285625
colsample_bytree	0.754514538349647
subsample	0.5019721075915964
learning_rate	0.08170979224393624
n_estimators	229
max_depth	3
min_child_weight	7

Table S6. Crystal data and structure refinement for $ABa_3(BSe_3)_2Cl$ (A= Rb, Cs).

Empirical formula	$RbBa_3(BSe_3)_2Cl$	$CsBa_3(BSe_3)_2Cl$
formula weight	1028.32	1075.76
temperature	245.0 K	245.0 K
crystal system, space group	orthorhombic, $Cmc2_1$	orthorhombic, *Pbca*
unit cell dimensions	a = 15.403(2) Å	a = 11.8928(8) Å
	b = 11.8262(18) Å	b = 8.6874(5) Å
	c = 8.6681(14) Å	c = 31.030(2) Å
volume	2551.80(18) $Å^3$	3206.0(4) $Å^3$
Z, calculated density	4, 4.326 g/cm^3	8, 4.458 g/cm^3
absorption coefficient	24.421 mm^{-1}	23.277 mm^{-1}
F(000)	1744.0	3632.0
2θ range for data collection	4.342 – 54.984 °	4.316 – 54.972 °
limiting indices	$-17 \leq h \leq 19, -15 \leq k \leq 15, -11 \leq l \leq 10$	$-15 \leq h \leq 14, -10 \leq k \leq 11, -40 \leq l \leq 40$
reflections collected/unique	5758/1857[R(int) = 0.1158]	22471/3649[R(int) = 0.0900]
refinement method	full-matrix least-squares on F^2	full-matrix least-squares on F^2
data/restraints/parameters	1857/1/65	3649/0/119
goodness-of-fit on F^2	0.968	1.041
final R indices $[I > 2\sigma(I)]^a$	R_1 = 0.0558, wR_2 = 0.1300	R_1 = 0.0393, wR_2 = 0.0866
R indices (all data)^a	R_1 = 0.0630, wR_2 = 0.1393	R_1 = 0.0515, wR_2 = 0.0927
largest diff. peak and hole	1.63 and −1.63 e·$Å^{-3}$	2.41 and −1.80 e·$Å^{-3}$
flack parameter	0.00(5)	/

$^aR_1 = \Sigma||F_o| - |F_c||/\Sigma|F_o|$ and $wR_2 = [\Sigma w(F_o^2 - F_c^2)^2/\Sigma wF_o^4]^{1/2}$ for $F_o^2 > 2\sigma(F_o^2)$

Table S7. Crystal data and structure refinement for $ABa_3(BSe_3)_2Br$ (A= Rb, Cs).

Empirical formula	$RbBa_3(BSe_3)_2Br$	$CsBa_3(BSe_3)_2Br$
formula weight	1072.78	1120.22
temperature	100.0 K	245.0 K
crystal system, space group	orthorhombic, $Cmc2_1$	orthorhombic, $Cmc2_1$
unit cell dimensions	a = 16.0147(11) Å	a = 16.1046(13) Å
	b = 11.6688(7) Å	b = 11.7634(10) Å
	c = 8.5898(5) Å	c = 8.5819(6) Å
volume	1605.20(17) $Å^3$	1625.8(2) $Å^3$
Z, calculated density	4, 4.439 g/cm^3	4, 4.577 g/cm^3
absorption coefficient	26.345 mm^{-1}	25.244 mm^{-1}
F(000)	1816.0	1888.0
2θ range for data collection	4.318 – 54.956 °	4.288 – 54.952 °
limiting indices	$-20 \le h \le 20$, $-15 \le k \le 15$, $-11 \le l \le 11$	$-20 \le h \le 20$, $-15 \le k \le 15$, $-11 \le l \le 11$
reflections collected/unique	5887/1837[R(int) = 0.0617]	6008/1888[R(int) = 0.0678]
refinement method	full-matrix least-squares on F^2	full-matrix least-squares on F^2
data/restraints/parameters	1837/1/65	1888/7/66
goodness-of-fit on F^2	1.011	1.045
final R indices [$I > 2\sigma(I)$][a]	R_1 = 0.0314, wR_2 = 0.0558	R_1 = 0.0341, wR_2 = 0.0651
R indices (all data)[a]	R_1 = 0.0334, wR_2 = 0.0566	R_1 = 0.0366, wR_2 = 0.0664
largest diff. peak and hole	1.80 and −1.03 e·Å^{-3}	1.91 and −2.33 e·Å^{-3}
flack parameter	−0.02(2)	−0.02(2)

[a] $R_1 = \Sigma||F_o| - |F_c||/\Sigma|F_o|$ and $wR_2 = [\Sigma w(F_o^2 - F_c^2)^2/\Sigma wF_o^4]^{1/2}$ for $F_o^2 > 2\sigma(F_o^2)$

Table S8. Crystal data and structure refinement for $ABa_3(BSe_3)_2I$ (A= Rb, Cs).

Empirical formula	$RbBa_3(BSe_3)_2I$	$CsBa_3(BSe_3)_2I$
formula weight	1119.77	1167.21
temperature	173.0 K	273.15 K
crystal system, space group	orthorhombic, $Cmc2_1$	orthorhombic, $Cmc2_1$
unit cell dimensions	a = 16.3038(19) Å	a = 16.3293(17) Å
	b = 11.7474(11) Å	b = 11.8362(11) Å
	c = 8.5726(8) Å	c = 8.5707(6) Å
volume	1641.9(3) $Å^3$	1656.5(3) $Å^3$
Z, calculated density	4, 4.530 g/cm^3	4, 4.680 g/cm^3
absorption coefficient	25.204 mm^{-1}	24.228 mm^{-1}
F(000)	1888.0	1960.0
2θ range for data collection	4.274 – 55.004 °	4.25 – 55.036 °
limiting indices	$-21 \leq h \leq 21, -13 \leq k \leq 15, -11 \leq l \leq 10$	$-21 \leq h \leq 21, -15 \leq k \leq 15, -11 \leq l \leq 10$
reflections collected/unique	6131/1727[R(int) = 0.0748]	6069/1886[R(int) = 0.0820]
refinement method	full-matrix least-squares on F^2	full-matrix least-squares on F^2
data/restraints/parameters	1727/7/65	1886/1/65
goodness-of-fit on F^2	1.079	0.963
final R indices [$I > 2\sigma(I)$][a]	R_1 = 0.0373, wR_2 = 0.0850	R_1 = 0.0381, wR_2 = 0.0736
R indices (all data)[a]	R_1 = 0.0393, wR_2 = 0.0866	R_1 = 0.0404, wR_2 = 0.0751
largest diff. peak and hole	2.33 and −1.41 e·$Å^{-3}$	1.34 and −1.08 e·$Å^{-3}$
flack parameter	−0.01(3)	0.04(3)

[a] $R_1 = \Sigma||F_o| - |F_c||/\Sigma|F_o|$ and $wR_2 = [\Sigma w(F_o^2 - F_c^2)^2/\Sigma wF_o^4]^{1/2}$ for $F_o^2 > 2\sigma(F_o^2)$

Table S9. Fractional atomic coordinates (× 10^4), equivalent isotropic displacement parameters (Å^2 × 10^3), and bond valence sum (BVS) for $RbBa_3(BSe_3)_2Cl$. U_{eq} is defined as 1/3 of the trace of the orthogonalized U_{IJ} tensor.

Atom	*x*	*y*	*z*	U_{eq}	BVS
Rb1	5000	5890(4)	5374(6)	48.4(12)	0.79
Ba1	7062.4(9)	3001.9(11)	6679.0(17)	18.2(3)	2.26
Ba2	10000	5272.3(16)	6741(2)	18.4(4)	2.03
B1	8060(20)	4660(30)	3980(30)	25(6)	2.97
Se1	6862.5(17)	4445(2)	3258(3)	24.8(6)	1.76
Se2	8609.0(17)	6181(2)	4050(3)	19.5(5)	2.08
Se3	8786.8(15)	3416(2)	4551(3)	18.5(5)	2.35
Cl1	5000	3251(7)	6543(12)	25.3(17)	0.91

Table S10. Fractional atomic coordinates (× 10^4), equivalent isotropic displacement parameters (Å^2 × 10^3), and bond valence sum (BVS) for $RbBa_3(BSe_3)_2Br$. U_{eq} is defined as 1/3 of the trace of the orthogonalized U_{IJ} tensor.

Atom	*x*	*y*	*z*	U_{eq}	BVS
Rb1	0	6413(2)	3119(3)	45.4(7)	0.87
Ba1	5000	4655.7(8)	7731.5(13)	16.0(2)	2.02
Ba2	2089.5(4)	3022.7(6)	2671.2(9)	16.15(16)	2.23
B1	3125(8)	5348(12)	4950(16)	16(3)	2.96
Se1	1998.3(8)	5581.0(11)	4212.1(17)	23.4(3)	2.02
Se2	3823.8(8)	6648.0(10)	5556.3(14)	16.9(3)	2.09
Se3	3654.5(8)	3817.0(10)	5025.9(15)	16.5(3)	2.08
Br1	0	3420.2(17)	2267(2)	28.1(5)	0.89

Table S11. Fractional atomic coordinates (× 10^4), equivalent isotropic displacement parameters (Å^2 × 10^3), and bond valence sum (BVS) for $RbBa_3(BSe_3)_2I$. U_{eq} is defined as 1/3 of the trace of the orthogonalized U_{IJ} tensor.

Atom	*x*	*y*	*z*	U_{eq}	BVS
Rb1	10000	1514(2)	7808(4)	29.3(7)	0.89
Ba1	10000	5332.4(11)	7581.6(18)	16.2(3)	2.03
Ba2	7141.3(5)	7000.3(8)	2528.7(14)	17.0(2)	2.25
B1	8153(11)	4684(14)	4780(20)	14(3)	2.98
Se1	8663.4(10)	6189.3(13)	4890(2)	17.4(4)	2.06
Se2	7019.1(11)	4458.0(15)	4100(2)	23.2(4)	1.86
Se3	8820.5(11)	3379.2(13)	5363.0(19)	17.6(4)	2.13
I1	5000	6517.7(14)	2169(2)	29.0(4)	1.19

Table S12. Fractional atomic coordinates (× 10^4), equivalent isotropic displacement parameters (Å^2 × 10^3), and bond valence sum (BVS) for $CsBa_3(BSe_3)_2Cl$. U_{eq} is defined as 1/3 of the trace of the orthogonalized U_{IJ} tensor.

**Atom**	*x*	*y*	*z*	U_{eq}	**BVS**
Cs1	1426.2(6)	6573.6(10)	3778.3(2)	43.1(2)	0.90
Ba1	4482.4(4)	7547.3(5)	2726.1(2)	15.91(13)	2.19
Ba2	4525.7(4)	7304.5(5)	4784.1(2)	16.06(13)	2.19
Ba3	7837.2(4)	2564.2(5)	3736.2(2)	15.74(13)	2.06
B1	2220(8)	9824(9)	2231(3)	14.1(18)	2.96
B2	7393(8)	5253(11)	4706(3)	19.1(19)	2.95
Se1	947.2(7)	10413.9(9)	1856.9(3)	16.39(19)	2.21
Se2	3699.3(7)	9888.4(10)	1957.1(3)	16.52(19)	2.14
Se3	1947.1(8)	9139.7(11)	2815.8(3)	24.3(2)	1.85
Se4	6128.5(7)	4681.5(9)	4336.1(3)	16.23(19)	2.21
Se5	7101.8(8)	5894.4(11)	5294.5(3)	25.1(2)	1.93
Se6	8895.1(7)	5201.7(10)	4436.1(3)	17.08(19)	2.02
Cl1	4293.4(19)	7483(2)	3755.5(6)	21.2(4)	0.88

Table S13. Fractional atomic coordinates (× 10^4), equivalent isotropic displacement parameters (Å^2 × 10^3), and bond valence sum (BVS) for $CsBa_3(BSe_3)_2Br$. U_{eq} is defined as 1/3 of the trace of the orthogonalized U_{IJ} tensor.

Atom	x	y	z	U(eq)	BVS
Cs1	0	3619.0(13)	2885(3)	42.6(5)	1.13
Ba1	5000	5349.2(9)	7700.6(16)	15.8(2)	2.02
Ba2	2090.0(4)	6991.4(6)	2648.9(11)	16.3(2)	2.17
B1	3118(9)	4692(12)	4932(18)	15(3)	2.97
Se1	3654.8(9)	6200.1(12)	4997.2(17)	17.7(3)	2.08
Se2	3811.6(10)	3396.6(12)	5518.8(17)	17.9(3)	2.15
Se3	1990.7(9)	4457.5(13)	4234(2)	25.1(4)	2.03
Br1	0	6674.2(18)	2310(3)	29.6(6)	0.93

Table S14. Fractional atomic coordinates (× 10^4), equivalent isotropic displacement parameters ($Å^2$ × 10^3), and bond valence sum (BVS) for $CsBa_3(BSe_3)_2I$. U_{eq} is defined as 1/3 of the trace of the orthogonalized U_{IJ} tensor.

Atom	x	y	z	U(eq)	BVS
Cs1	5000	8498.3(14)	7255(3)	36.8(5)	1.23
Ba1	5000	5323.8(10)	2406.2(17)	16.4(3)	2.03
Ba2	7856.8(5)	7006.8(7)	7453.9(13)	16.9(2)	2.21
B1	6866(11)	4719(15)	5190(20)	16(4)	2.98
Se1	6337.9(10)	6197.9(13)	5092(2)	17.4(4)	2.12
Se2	6189.3(11)	3409.3(13)	4635.0(17)	17.5(4)	2.15
Se3	7988.0(11)	4478.2(15)	5865(2)	24.4(4)	1.91
I1	10000	6581.1(13)	7764.0(19)	27.0(4)	1.1

Table S15. Selected bond lengths [Å] for $RbBa_3(BSe_3)_2Cl$.

Atom	Atom	Length/Å	Atom	Atom	Length/Å
Rb1	Cl1[10]	3.472(12)	Ba2	Se2[4]	3.345(3)
Rb1	Cl1	3.282(10)	Ba2	Se2[1]	3.399(3)
Rb1	Se1	3.810(4)	Ba2	Se2[5]	3.399(3)
Rb1[9]	Se3	3.594(5)	Ba2	Se2	3.345(3)
Rb1[10]	Se1	3.826(4)	Ba2	Se3	3.452(3)
Ba1	Se2[5]	3.291(3)	Ba2	Se3[5]	3.440(3)
Ba1	Se2[8]	3.301(3)	Ba2	Se3[1]	3.440(3)
Ba1	Se3[7]	3.274(3)	Ba2	Se3[4]	3.452(3)
Ba1	Se3	3.271(3)	Ba2	Cl1[3]	3.527(9)
Ba1	Se1	3.435(3)	B1	Se2	1.99(3)
Ba1	Se1[5]	3.329(3)	B1	Se3	1.91(3)
Ba1	Se1[7]	3.605(3)	B1	Se1	1.97(3)
Ba1	Cl1	3.1924(17)			

[1]2-X,1-Y,1/2+Z; [2]2-X,1-Y,-1/2+Z; [3]1/2+X,1/2+Y,+Z; [4]2-X,+Y,+Z; [5]+X,1-Y,1/2+Z; [6]3/2-X,1/2-Y,-1/2+Z; [7]3/2-X,1/2-Y,1/2+Z; [8]3/2-X,-1/2+Y,+Z; [9]1/2+X,-1/2+Y,+Z; [10]1-X,1-Y,-1/2+Z

Table S16. Selected bond lengths [Å] for $RbBa_3(BSe_3)_2Br$.

Atom	Atom	Length/Å	Atom	Atom	Length/Å
Rb1	Se1	3.4736(15)	Ba1	$Br1^{3}$	3.611(2)
Rb1	Br1	3.569(3)	Ba2	$Se3^{6}$	3.3453(14)
$Rb1^{9}$	Se3	3.898(3)	Ba2	Se3	3.3513(14)
$Rb1^{10}$	Se2	3.676(2)	Ba2	$Se2^{7}$	3.3409(14)
$Rb1^{11}$	Br1	3.568(3)	Ba2	$Se2^{8}$	3.2945(14)
Ba1	Se3	3.3170(15)	Ba2	Br1	3.3961(7)
Ba1	$Se3^{4}$	3.4211(15)	Ba2	$Se1^{7}$	3.3919(15)
Ba1	$Se3^{1}$	3.4211(15)	Ba2	Se1	3.2688(14)
Ba1	$Se3^{5}$	3.3170(15)	Ba2	$Se1^{8}$	3.4646(15)
Ba1	$Se2^{5}$	3.5276(15)	B1	Se1	1.931(13)
Ba1	$Se2^{4}$	3.4278(15)	B1	Se2	1.956(13)
Ba1	$Se2^{1}$	3.4278(15)	B1	Se3	1.978(13)
Ba1	Se2	3.5276(15)			

^{1}1-X,1-Y,1/2+Z; ^{2}1-X,1-Y,-1/2+Z; ^{3}1/2-X,1/2-Y,1/2+Z; ^{4}+X,1-Y,1/2+Z; ^{5}1-X,+Y,+Z; ^{6}1/2-X,1/2-Y,-1/2+Z; ^{7}+X,1-Y,-1/2+Z; ^{8}1/2-X,-1/2+Y,+Z; ^{9}1/2+X,-1/2+Y,+Z; ^{10}1/2-X,3/2-Y,1/2+Z; ^{11}-X,1-Y,-1/2+Z

Table S17. Selected bond lengths [Å] for $RbBa_3(BSe_3)_2I$.

Atom	Atom	Length/Å	Atom	Atom	Length/Å
Rb1	$Se1^{1}$	3.900(3)	Ba1	Se3	3.547(2)
Rb1	$Se1^{5}$	3.900(3)	Ba1	$Se3^{5}$	3.417(2)
Rb1	Se3	3.591(3)	Ba1	$Se3^{1}$	3.417(2)
Rb1	$Se3^{4}$	3.591(3)	Ba2	I1	3.5502(10)
Rb1	$Se2^{11}$	3.656(2)	Ba2	$Se1^{6}$	3.370(2)
Rb1	$Se2^{12}$	3.656(2)	Ba2	Se1	3.341(2)
$Rb1^{9}$	I1	3.738(4)	Ba2	$Se3^{7}$	3.315(2)
$Rb1^{10}$	I1	3.603(3)	Ba2	$Se3^{8}$	3.338(2)
Ba1	$I1^{3}$	3.717(2)	Ba2	$Se2^{8}$	3.408(2)
Ba1	$Se1^{4}$	3.329(2)	Ba2	Se2	3.282(2)
Ba1	Se1	3.329(2)	Ba2	$Se2^{7}$	3.468(2)
Ba1	$Se1^{5}$	3.444(2)	B1	Se1	1.957(17)
Ba1	$Se1^{1}$	3.444(2)	B1	Se2	1.957(18)
Ba1	$Se3^{4}$	3.547(2)	B1	Se3	1.945(18)

^{1}2-X,1-Y,1/2+Z; ^{2}2-X,1-Y,-1/2+Z; ^{3}3/2-X,3/2-Y,1/2+Z; ^{4}2-X,+Y,+Z; ^{5}+X,1-Y,1/2+Z; ^{6}3/2-X,3/2-Y,-1/2+Z; ^{7}3/2-X,1/2+Y,+Z; ^{8}+X,1-Y,-1/2+Z; ^{9}-1/2+X,1/2+Y,-1+Z; ^{10}3/2-X,1/2-Y,-1/2+Z; ^{11}1/2+X,1/2-Y,1/2+Z; ^{12}3/2-X,1/2-Y,1/2+Z

Table S18. Selected bond lengths [Å] for $CsBa_3(BSe_3)_2Cl$.

Atom	Atom	Length/Å	Atom	Atom	Length/Å
Cs1	$Se1^{11}$	3.5871(11)	Ba2	Se5	3.6600(11)
Cs1	$Se6^{12}$	3.8273(11)	Ba2	$Se5^{5}$	3.3953(10)
Cs1	Se3	3.7781(12)	Ba2	$Se5^{7}$	3.2890(10)
Cs1	$Se3^{10}$	4.1394(13)	Ba2	Cl1	3.207(2)
Cs1	$Se5^{7}$	3.7097(12)	Ba3	Se4	3.3134(9)
Cs1	$Se5^{5}$	3.9925(13)	Ba3	$Se4^{2}$	3.3540(10)
Cs1	Cl1	3.501(2)	Ba3	$Se1^{3}$	3.4068(10)
Cs1	$Cl1^{10}$	3.655(2)	Ba3	$Se1^{4}$	3.4540(10)
Ba1	$Se1^{9}$	3.3030(10)	Ba3	Se6	3.3985(10)
Ba1	$Se1^{10}$	3.3121(10)	Ba3	$Se6^{2}$	3.6295(10)
Ba1	Se2	3.2707(9)	Ba3	$Se2^{4}$	3.3292(10)
Ba1	$Se2^{3}$	3.3133(10)	Ba3	$Se2^{3}$	3.4706(10)
Ba1	Se3	3.3290(10)	Ba3	$Cl1^{2}$	3.414(2)
Ba1	$Se3^{9}$	3.6514(11)	B1	Se1	1.976(10)
Ba1	$Se3^{10}$	3.4251(10)	B1	Se2	1.955(9)
Ba1	Cl1	3.203(2)	B1	Se3	1.936(9)
Ba2	Se4	3.2799(9)	B2	Se4	1.956(10)
Ba2	$Se4^{5}$	3.3220(10)	B2	Se6	1.973(10)
Ba2	$Se6^{1}$	3.3209(10)	B2	Se5	1.941(10)
Ba2	$Se6^{7}$	3.3334(10)			

[1]3/2-X,1/2+Y,+Z; [2]3/2-X,-1/2+Y,+Z; [3]1-X,-1/2+Y,1/2-Z; [4]1/2+X,-1+Y,1/2-Z; [5]1-X,1-Y,1-Z; [6]1/2-X,1/2+Y,+Z; [7]-1/2+X,3/2-Y,1-Z; [8]1-X,1/2+Y,1/2-Z; [9]1/2+X,+Y,1/2-Z; [10]1/2-X,-1/2+Y,+Z; [11]-X,-1/2+Y,1/2-Z; [12]-1+X,+Y,+Z

Table S19. Selected bond lengths [Å] for $CsBa_3(BSe_3)_2Br$.

Atom	Atom	Length/Å	Atom	Atom	Length/Å
Cs1	$Se1^{10}$	4.010(2)	Ba1	$Se2^{4}$	3.4189(18)
Cs1	$Se1^{11}$	4.010(2)	Ba1	$Se2^{5}$	3.5278(18)
Cs1	$Se2^{12}$	3.662(2)	Ba1	$Br1^{6}$	3.518(2)
Cs1	$Se2^{13}$	3.662(2)	Ba2	$Se1^{7}$	3.3382(17)
Cs1	$Br1^{14}$	3.813(3)	Ba2	Se1	3.3583(17)
Cs1	Br1	3.628(3)	Ba2	$Se2^{8}$	3.3521(18)
Cs1	$Se3^{15}$	3.5485(17)	Ba2	$Se2^{9}$	3.3025(17)
Cs1	Se3	3.5486(17)	Ba2	Br1	3.3990(8)
Ba1	$Se1^{2}$	3.4496(17)	Ba2	$Se3^{8}$	3.3936(18)
Ba1	$Se1^{4}$	3.4496(17)	Ba2	Se3	3.2805(16)
Ba1	$Se1^{5}$	3.3284(18)	Ba2	$Se3^{9}$	3.5297(18)
Ba1	Se1	3.3284(18)	B1	Se1	1.974(14)
Ba1	$Se2^{2}$	3.4189(18)	B1	Se2	1.955(15)
Ba1	Se2	3.5277(18)	B1	Se3	1.932(15)

^{1}1-X,1-Y,-1/2+Z; ^{2}1-X,1-Y,1/2+Z; ^{3}1/2+X,3/2-Y,1/2+Z; ^{4}+X,1-Y,1/2+Z; ^{5}1-X,+Y,+Z; ^{6}1/2-X,3/2-Y,1/2+Z; ^{7}1/2-X,3/2-Y,-1/2+Z; ^{8}+X,1-Y,-1/2+Z; ^{9}1/2-X,1/2+Y,+Z; ^{10}1/2-X,-1/2+Y,+Z; ^{11}-1/2+X,-1/2+Y,+Z; ^{12}1/2-X,1/2-Y,-1/2+Z; ^{13}-1/2+X,1/2-Y,-1/2+Z; ^{14}-X,1-Y,1/2+Z; ^{15}-X,+Y,+Z

Table S20. Selected bond lengths [Å] for $CsBa_3(BSe_3)_2I$.

Atom	Atom	Length/Å	Atom	Atom	Length/Å
Cs1	Se1	3.952(2)	Ba1	Se2	3.543(2)
Cs1	Se1^{5}	3.952(2)	Ba1	Se2^{5}	3.543(2)
Cs1	Se2^{2}	3.610(2)	Ba1	Se2^{4}	3.4148(19)
Cs1	Se2^{8}	3.610(2)	Ba2	I1	3.5458(10)
Cs1	Se3^{7}	3.6822(19)	Ba2	Se1	3.3412(19)
Cs1	Se3^{11}	3.6822(19)	Ba2	Se1^{6}	3.3703(19)
Cs1^{6}	I1	3.850(3)	Ba2	Se2^{7}	3.3195(19)
Cs1^{9}	I1	3.675(2)	Ba2	Se2^{8}	3.339(2)
Ba1	I1^{3}	3.676(2)	Ba2	Se3^{7}	3.509(2)
Ba1	Se1^{1}	3.457(2)	Ba2	Se3	3.2950(19)
Ba1	Se1	3.338(2)	Ba2	Se3^{8}	3.418(2)
Ba1	Se1^{4}	3.457(2)	B1	Se1	1.953(17)
Ba1	Se1^{5}	3.338(2)	B1	Se2	1.962(18)
Ba1	Se2^{1}	3.4148(19)	B1	Se3	1.943(18)

^{1}1-X,1-Y,-1/2+Z; ^{2}1-X,1-Y,1/2+Z; ^{3}3/2-X,3/2-Y,-1/2+Z; ^{4}+X,1-Y,-1/2+Z; ^{5}1-X,+Y,+Z; ^{6}3/2-X,3/2-Y,1/2+Z; ^{7}3/2-X,1/2+Y,+Z; ^{8}+X,1-Y,1/2+Z; ^{9}1/2+X,-1/2+Y,+Z; ^{10}1-X,2-Y,1/2+Z; ^{11}-1/2+X,1/2+Y,+Z

Table S21. Selected bond angles [°] for $RbBa_3(BSe_3)_2Cl$.

Atom	Atom	Atom	Angle/°	Atom	Atom	Atom	Angle/°
$Se3^{10}$	Rb1	$Se3^{15}$	62.65(11)	$Se3^{7}$	Ba1	Se1	151.04(7)
$Se3^{15}$	Rb1	$Se1^{14}$	83.44(8)	Se3	Ba1	$Se1^{5}$	99.87(7)
$Se3^{10}$	Rb1	$Se1^{5}$	79.99(8)	$Se3^{7}$	Ba1	$Se1^{5}$	96.59(8)
$Se3^{15}$	Rb1	$Se1^{5}$	127.29(15)	$Se3^{7}$	Ba1	$Se1^{7}$	58.91(6)
$Se3^{15}$	Rb1	Se1	131.96(14)	Se3	Ba1	Se1	60.76(7)
$Se3^{10}$	Rb1	Se1	83.44(8)	$Se1^{5}$	Ba1	Se1	84.03(4)
$Se3^{15}$	Rb1	$Se1^{16}$	79.99(8)	Se1	Ba1	$Se1^{7}$	140.17(8)
$Se3^{10}$	Rb1	$Se1^{14}$	131.96(14)	$Se1^{5}$	Ba1	$Se1^{7}$	127.91(9)
$Se3^{10}$	Rb1	$Se1^{16}$	127.29(15)	Cl1	Ba1	$Se2^{9}$	73.92(18)
$Se1^{14}$	Rb1	$Se1^{5}$	146.37(13)	Cl1	Ba1	$Se2^{5}$	135.74(19)
$Se1^{14}$	Rb1	$Se1^{16}$	72.74(6)	Cl1	Ba1	$Se3^{7}$	71.19(18)
Se1	Rb1	$Se1^{16}$	146.37(13)	Cl1	Ba1	Se3	140.67(19)
Se1	Rb1	$Se1^{5}$	72.74(6)	Cl1	Ba1	Se1	80.40(19)
Se1	Rb1	$Se1^{14}$	97.71(15)	Cl1	Ba1	$Se1^{7}$	123.03(17)
$Se1^{5}$	Rb1	$Se1^{16}$	97.15(14)	Cl1	Ba1	$Se1^{5}$	80.77(17)
Cl1	Rb1	$Se3^{15}$	148.38(6)	Se2	Ba2	$Se2^{4}$	79.67(10)
Cl1	Rb1	$Se3^{10}$	148.38(6)	$Se2^{4}$	Ba2	$Se2^{5}$	167.70(8)
$Cl1^{13}$	Rb1	$Se3^{10}$	64.35(15)	Se2	Ba2	$Se2^{2}$	167.71(8)
$Cl1^{13}$	Rb1	$Se3^{15}$	64.35(15)	$Se2^{5}$	Ba2	$Se2^{2}$	78.16(10)
Cl1	Rb1	Se1	73.86(15)	Se2	Ba2	$Se2^{5}$	99.75(7)
$Cl1^{13}$	Rb1	$Se1^{5}$	130.94(7)	$Se2^{4}$	Ba2	$Se2^{2}$	99.75(7)
$Cl1^{13}$	Rb1	Se1	70.79(13)	$Se2^{5}$	Ba2	$Se3^{4}$	110.09(8)
Cl1	Rb1	$Se1^{14}$	73.86(15)	$Se2^{2}$	Ba2	Se3	110.09(8)
$Cl1^{13}$	Rb1	$Se1^{14}$	70.79(13)	$Se2^{4}$	Ba2	$Se3^{4}$	58.27(7)
Cl1	Rb1	$Se1^{5}$	72.51(14)	$Se2^{2}$	Ba2	$Se3^{5}$	98.84(8)
Cl1	Rb1	$Se1^{16}$	72.51(14)	$Se2^{4}$	Ba2	Se3	99.65(8)
$Cl1^{13}$	Rb1	$Se1^{16}$	130.94(7)	$Se2^{4}$	Ba2	$Se3^{2}$	90.05(6)
Cl1	Rb1	$Cl1^{13}$	125.0(3)	$Se2^{5}$	Ba2	Se3	70.18(6)
$Se2^{5}$	Ba1	$Se2^{9}$	148.12(5)	$Se2^{2}$	Ba2	$Se3^{4}$	70.18(6)
$Se2^{5}$	Ba1	$Se1^{7}$	70.49(7)	Se2	Ba2	$Se3^{2}$	134.17(8)
$Se2^{9}$	Ba1	Se1	72.55(7)	Se2	Ba2	$Se3^{4}$	99.65(8)
$Se2^{5}$	Ba1	Se1	117.26(7)	$Se2^{5}$	Ba2	$Se3^{2}$	98.84(8)
$Se2^{5}$	Ba1	$Se1^{5}$	62.87(7)	$Se2^{4}$	Ba2	$Se3^{5}$	134.18(8)
$Se2^{9}$	Ba1	$Se1^{5}$	147.82(8)	$Se2^{5}$	Ba2	$Se3^{5}$	57.91(6)
$Se2^{9}$	Ba1	$Se1^{7}$	83.24(7)	$Se2^{2}$	Ba2	$Se3^{2}$	57.91(7)
Se3	Ba1	$Se2^{5}$	73.77(7)	Se2	Ba2	$Se3^{5}$	90.05(6)
$Se3^{7}$	Ba1	$Se2^{5}$	87.98(7)	Se2	Ba2	Se3	58.27(7)
$Se3^{7}$	Ba1	$Se2^{9}$	93.77(7)	$Se2^{5}$	Ba2	$Cl1^{3}$	122.26(13)
Se3	Ba1	$Se2^{9}$	87.86(7)	$Se2^{2}$	Ba2	$Cl1^{3}$	122.26(13)
Se3	Ba1	$Se3^{7}$	146.09(6)	$Se2^{4}$	Ba2	$Cl1^{3}$	69.21(14)
Se3	Ba1	$Se1^{7}$	87.78(7)	Se2	Ba2	$Cl1^{3}$	69.21(14)

Se3	Ba2	Se3[4]	65.55(8)	Se3[5]	Ba2	Cl1[3]	65.41(15)
Se3[2]	Ba2	Se3	166.02(8)	Se3[2]	Ba2	Cl1[3]	65.41(15)
Se3[5]	Ba2	Se3	112.47(7)	Se3[4]	Ba2	Cl1[3]	127.49(14)
Se3[5]	Ba2	Se3[4]	166.02(8)	Se3	B1	Se2	116.2(15)
Se3[2]	Ba2	Se3[5]	65.82(9)	Se3	B1	Se1	122.1(16)
Se3[2]	Ba2	Se3[4]	112.47(7)	Se1	B1	Se2	121.6(16)
Se3	Ba2	Cl1[3]	127.49(14)				

[1]2-X,1-Y,-1/2+Z; [2]2-X,1-Y,1/2+Z; [3]1/2+X,1/2+Y,+Z; [4]2-X,+Y,+Z; [5]+X,1-Y,1/2+Z; [6]-1/2+X,-1/2+Y,+Z; [7]3/2-X,1/2-Y,1/2+Z; [8]3/2-X,1/2-Y,-1/2+Z; [9]3/2-X,-1/2+Y,+Z; [10]3/2-X,1/2+Y,+Z; [11]+X,1-Y,-1/2+Z; [12]1/2+X,-1/2+Y,+Z; [13]1-X,1-Y,-1/2+Z; [14]1-X,+Y,+Z; [15]-1/2+X,1/2+Y,+Z; [16]1-X,1-Y,1/2+Z

Table S22. Selected bond angles [°] for $RbBa_3(BSe_3)_2Br$.

Atom	Atom	Atom	Angle/°	Atom	Atom	Atom	Angle/°
$Se3^{11}$	Rb1	$Se3^{15}$	67.13(5)	$Se3^{5}$	Ba1	Se2	68.01(3)
$Se2^{16}$	Rb1	$Se3^{15}$	61.68(4)	$Se3^{4}$	Ba1	$Se2^{4}$	58.43(3)
$Se2^{16}$	Rb1	$Se3^{11}$	95.29(6)	$Se3^{4}$	Ba1	Se2	99.80(4)
$Se2^{14}$	Rb1	$Se3^{11}$	61.68(4)	$Se3^{2}$	Ba1	$Se2^{2}$	58.49(3)
$Se2^{14}$	Rb1	$Se3^{15}$	95.29(6)	Se3	Ba1	$Se2^{5}$	90.46(3)
$Se2^{16}$	Rb1	$Se2^{14}$	61.65(5)	$Se3^{2}$	Ba1	$Se2^{5}$	99.76(4)
$Br1^{17}$	Rb1	$Se3^{15}$	62.70(5)	$Se3^{5}$	Ba1	$Se2^{2}$	99.76(4)
Br1	Rb1	$Se3^{15}$	142.23(4)	$Se3^{4}$	Ba1	$Se2^{5}$	136.22(4)
Br1	Rb1	$Se3^{11}$	142.23(4)	$Se3^{4}$	Ba1	$Se2^{2}$	90.46(3)
$Br1^{17}$	Rb1	$Se3^{11}$	62.70(5)	$Se3^{2}$	Ba1	$Br1^{3}$	125.55(4)
$Br1^{17}$	Rb1	$Se2^{16}$	124.37(7)	$Se3^{5}$	Ba1	$Br1^{3}$	125.55(4)
$Br1^{17}$	Rb1	$Se2^{14}$	124.37(7)	Se3	Ba1	$Br1^{3}$	68.24(3)
Br1	Rb1	$Se2^{16}$	118.65(7)	$Se3^{4}$	Ba1	$Br1^{3}$	68.24(3)
Br1	Rb1	$Se2^{14}$	118.65(7)	$Se2^{4}$	Ba1	Se2	64.55(4)
$Br1^{17}$	Rb1	Br1	104.96(7)	$Se2^{2}$	Ba1	$Se2^{5}$	66.67(5)
$Se1^{12}$	Rb1	$Se3^{11}$	126.66(7)	$Se2^{2}$	Ba1	$Se2^{4}$	111.96(4)
$Se1^{12}$	Rb1	$Se3^{15}$	65.06(4)	$Se2^{5}$	Ba1	Se2	111.96(4)
Se1	Rb1	$Se3^{15}$	126.66(7)	$Se2^{2}$	Ba1	Se2	163.91(4)
Se1	Rb1	$Se3^{11}$	65.06(4)	$Se2^{5}$	Ba1	$Se2^{4}$	163.91(4)
Se1	Rb1	$Se2^{16}$	143.71(6)	$Se2^{4}$	Ba1	$Br1^{3}$	126.62(4)
$Se1^{12}$	Rb1	$Se2^{16}$	82.06(4)	$Se2^{2}$	Ba1	$Br1^{3}$	68.72(4)
$Se1^{12}$	Rb1	$Se2^{14}$	143.71(6)	Se2	Ba1	$Br1^{3}$	126.62(4)
Se1	Rb1	$Se2^{14}$	82.06(4)	$Se2^{5}$	Ba1	$Br1^{3}$	68.72(4)
$Se1^{12}$	Rb1	$Br1^{17}$	75.25(5)	$Se3^{6}$	Ba2	Se3	148.63(2)
Se1	Rb1	Br1	77.39(5)	Se3	Ba2	Br1	139.54(5)
$Se1^{12}$	Rb1	Br1	77.39(5)	$Se3^{6}$	Ba2	Br1	70.56(4)
Se1	Rb1	$Br1^{17}$	75.25(5)	$Se3^{6}$	Ba2	$Se1^{7}$	83.22(3)
Se1	Rb1	$Se1^{12}$	134.23(9)	Se3	Ba2	$Se1^{7}$	71.43(3)
$Se3^{4}$	Ba1	Se3	81.03(5)	Se3	Ba2	$Se1^{8}$	115.34(4)
Se3	Ba1	$Se3^{2}$	165.14(4)	$Se3^{6}$	Ba2	$Se1^{8}$	72.41(3)
$Se3^{4}$	Ba1	$Se3^{5}$	165.14(4)	$Se2^{8}$	Ba2	Se3	71.02(3)
$Se3^{2}$	Ba1	$Se3^{5}$	78.08(5)	$Se2^{7}$	Ba2	Se3	90.73(4)
$Se3^{4}$	Ba1	$Se3^{2}$	98.52(4)	$Se2^{8}$	Ba2	$Se3^{6}$	90.03(3)
Se3	Ba1	$Se3^{5}$	98.52(3)	$Se2^{7}$	Ba2	$Se3^{6}$	92.31(3)
$Se3^{2}$	Ba1	Se2	107.35(4)	$Se2^{7}$	Ba2	$Se2^{8}$	146.52(3)
$Se3^{5}$	Ba1	$Se2^{4}$	107.35(4)	$Se2^{8}$	Ba2	Br1	138.40(5)
Se3	Ba1	Se2	58.43(3)	$Se2^{7}$	Ba2	Br1	72.90(4)
$Se3^{5}$	Ba1	$Se2^{5}$	58.49(3)	$Se2^{8}$	Ba2	$Se1^{7}$	87.24(3)
Se3	Ba1	$Se2^{4}$	99.80(4)	$Se2^{8}$	Ba2	$Se1^{8}$	60.28(3)
Se3	Ba1	$Se2^{2}$	136.22(4)	$Se2^{7}$	Ba2	$Se1^{8}$	150.89(4)
$Se3^{2}$	Ba1	$Se2^{4}$	68.01(3)	$Se2^{7}$	Ba2	$Se1^{7}$	59.96(3)

Br1	Ba2	Se1[7]	124.53(4)	Se1	Ba2	Se1[7]	127.96(5)
Se1	Ba2	Se3[6]	147.69(4)	Se1[8]	Ba2	Se1[7]	138.40(4)
Se1	Ba2	Se3	62.38(3)	Se1	Ba2	Se1[8]	85.07(2)
Se1	Ba2	Se2[7]	96.91(4)	Se2	B1	Se3	116.5(6)
Se1	Ba2	Se2[8]	98.76(4)	Se1	B1	Se3	122.6(7)
Se1[8]	Ba2	Br1	78.60(4)	Se1	B1	Se2	120.8(7)
Se1	Ba2	Br1	82.68(4)				

[1]1-X,1-Y,-1/2+Z; [2]1-X,1-Y,1/2+Z; [3]1/2-X,1/2-Y,1/2+Z; [4]1-X,+Y,+Z; [5]+X,1-Y,1/2+Z; [6]1/2-X,1/2-Y,-1/2+Z; [7]1/2-X,-1/2+Y,+Z; [8]+X,1-Y,-1/2+Z; [9]1/2+X,-1/2+Y,+Z; [10]1/2-X,3/2-Y,1/2+Z; [11]1/2-X,1/2+Y,+Z; [12]-X,+Y,+Z; [13]-X,1-Y,-1/2+Z; [14]1/2-X,3/2-Y,-1/2+Z; [15]-1/2+X,1/2+Y,+Z; [16]-1/2+X,3/2-Y,-1/2+Z; [17]-X,1-Y,1/2+Z

Table S23. Selected bond angles [°] for $RbBa_3(BSe_3)_2I$.

Atom	Atom	Atom	Angle/°	Atom	Atom	Atom	Angle/°
I1^{13}	Rb1	I1^{12}	98.81(7)	Se1^{5}	Ba1	Se3	100.30(6)
I1^{12}	Rb1	Se1^{1}	62.72(6)	Se1^{4}	Ba1	Se3^{5}	108.39(5)
I1^{13}	Rb1	Se1^{4}	138.87(5)	Se1	Ba1	Se3^{5}	100.30(6)
I1^{12}	Rb1	Se1^{4}	62.72(6)	Se1	Ba1	Se3^{1}	135.89(6)
I1^{13}	Rb1	Se1^{1}	138.87(5)	Se1^{5}	Ba1	Se3^{1}	88.93(4)
I1^{13}	Rb1	Se2^{14}	74.77(5)	Se1^{5}	Ba1	Se3^{5}	57.94(4)
I1^{13}	Rb1	Se2^{13}	74.77(5)	Se1^{1}	Ba1	Se3	108.39(5)
Se1^{1}	Rb1	Se1^{4}	67.93(7)	Se1^{1}	Ba1	Se3^{5}	68.24(4)
Se3^{5}	Rb1	I1^{13}	120.95(8)	Se1	Ba1	Se3	57.94(4)
Se3	Rb1	I1^{13}	120.95(8)	Se3^{4}	Ba1	I1^{3}	68.00(4)
Se3	Rb1	I1^{12}	125.67(7)	Se3^{1}	Ba1	I1^{3}	68.00(4)
Se3^{5}	Rb1	I1^{12}	125.67(7)	Se3^{5}	Ba1	I1^{3}	126.37(5)
Se3	Rb1	Se1^{1}	98.30(7)	Se3	Ba1	I1^{3}	126.37(5)
Se3	Rb1	Se1^{4}	62.99(5)	Se3^{1}	Ba1	Se1^{1}	58.18(4)
Se3^{5}	Rb1	Se1^{4}	98.30(7)	Se3^{1}	Ba1	Se1^{4}	100.66(6)
Se3^{5}	Rb1	Se1^{1}	62.99(5)	Se3^{4}	Ba1	Se1^{1}	100.66(6)
Se3^{5}	Rb1	Se3	64.76(7)	Se3^{4}	Ba1	Se1^{4}	58.18(5)
Se3^{5}	Rb1	Se2^{13}	148.17(8)	Se3^{4}	Ba1	Se3^{5}	165.00(6)
Se3	Rb1	Se2^{14}	148.17(8)	Se3^{1}	Ba1	Se3	165.00(6)
Se3	Rb1	Se2^{13}	83.41(5)	Se3	Ba1	Se3^{5}	65.66(6)
Se3^{5}	Rb1	Se2^{14}	83.41(5)	Se3^{1}	Ba1	Se3^{4}	68.50(7)
Se2^{13}	Rb1	I1^{12}	72.39(6)	Se3^{4}	Ba1	Se3	110.83(5)
Se2^{14}	Rb1	I1^{12}	72.39(6)	Se3^{1}	Ba1	Se3^{5}	110.83(5)
Se2^{13}	Rb1	Se1^{4}	64.81(5)	Se1^{6}	Ba2	I1	70.11(4)
Se2^{14}	Rb1	Se1^{4}	125.48(9)	Se1	Ba2	I1	137.51(5)
Se2^{14}	Rb1	Se1^{1}	64.81(5)	Se1	Ba2	Se1^{6}	151.12(3)
Se2^{13}	Rb1	Se1^{1}	125.48(9)	Se1	Ba2	Se2^{7}	115.00(5)
Se2^{14}	Rb1	Se2^{13}	128.41(10)	Se1^{6}	Ba2	Se2^{7}	73.48(5)
Se1^{1}	Ba1	I1^{3}	124.85(4)	Se1	Ba2	Se2^{8}	73.07(5)
Se1	Ba1	I1^{3}	68.47(4)	Se1^{6}	Ba2	Se2^{8}	83.63(5)
Se1^{5}	Ba1	I1^{3}	68.47(4)	Se3^{8}	Ba2	I1	71.12(5)
Se1^{4}	Ba1	I1^{3}	124.85(4)	Se3^{7}	Ba2	I1	137.47(5)
Se1^{1}	Ba1	Se1^{4}	78.50(7)	Se3^{8}	Ba2	Se1	92.67(5)
Se1	Ba1	Se1^{1}	165.76(5)	Se3^{7}	Ba2	Se1	71.93(5)
Se1	Ba1	Se1^{5}	81.77(7)	Se3^{7}	Ba2	Se1^{6}	91.74(5)
Se1^{5}	Ba1	Se1^{4}	165.76(5)	Se3^{8}	Ba2	Se1^{6}	89.97(5)
Se1	Ba1	Se1^{4}	98.11(5)	Se3^{8}	Ba2	Se3^{7}	149.43(4)
Se1^{5}	Ba1	Se1^{1}	98.11(5)	Se3^{7}	Ba2	Se2^{7}	60.07(5)
Se1^{4}	Ba1	Se3	68.24(4)	Se3^{8}	Ba2	Se2^{7}	148.24(5)
Se1^{5}	Ba1	Se3^{4}	135.89(6)	Se3^{8}	Ba2	Se2^{8}	59.67(5)
Se1	Ba1	Se3^{4}	88.93(4)	Se3^{7}	Ba2	Se2^{8}	90.20(5)

Se2[7]	Ba2	I1	77.72(5)	Se2	Ba2	Se2[7]	83.86(3)
Se2	Ba2	I1	80.25(5)	Se2[7]	Ba2	Se2[8]	140.96(6)
Se2[8]	Ba2	I1	123.70(5)	Se2	Ba2	Se2[8]	128.46(7)
Se2	Ba2	Se1[6]	145.68(5)	Se1	B1	Se2	122.6(9)
Se2	Ba2	Se1	62.42(5)	Se3	B1	Se1	117.5(9)
Se2	Ba2	Se3[8]	96.61(5)	Se3	B1	Se2	119.9(8)
Se2	Ba2	Se3[7]	99.01(5)				

[1]2-X,1-Y,1/2+Z; [2]2-X,1-Y,-1/2+Z; [3]3/2-X,3/2-Y,1/2+Z; [4]+X,1-Y,1/2+Z; [5]2-X,+Y,+Z; [6]3/2-X,3/2-Y,-1/2+Z; [7]+X,1-Y,-1/2+Z; [8]3/2-X,1/2+Y,+Z; [9]-1/2+X,1/2+Y,-1+Z; [10]1-X,+Y,+Z; [11]3/2-X,1/2-Y,-1/2+Z; [12]1/2+X,-1/2+Y,1+Z; [13]3/2-X,1/2-Y,1/2+Z; [14]1/2+X,1/2-Y,1/2+Z; [15]3/2-X,-1/2+Y,+Z

Table S24. Selected bond angles [°] for $CsBa_3(BSe_3)_2Cl$.

Atom	Atom	Atom	Angle/°	Atom	Atom	Atom	Angle/°
$Se1^{11}$	Cs1	$Se6^{12}$	65.59(2)	$Se2^{4}$	Ba1	$Se3^{10}$	72.38(2)
$Se1^{11}$	Cs1	Se3	81.97(3)	$Se2^{4}$	Ba1	$Se3^{9}$	82.93(2)
$Se1^{11}$	Cs1	$Se3^{10}$	80.08(2)	$Se2^{4}$	Ba1	Se3	148.87(3)
$Se1^{11}$	Cs1	$Se5^{6}$	126.17(3)	Se2	Ba1	$Se3^{10}$	117.09(3)
$Se1^{11}$	Cs1	$Se5^{7}$	139.74(3)	Se2	Ba1	$Se3^{9}$	69.94(2)
$Se1^{11}$	Cs1	$Cl1^{10}$	62.10(4)	Se3	Ba1	$Se3^{10}$	84.422(15)
$Se6^{12}$	Cs1	$Se3^{10}$	126.40(3)	$Se3^{10}$	Ba1	$Se3^{9}$	139.76(3)
$Se6^{12}$	Cs1	$Se5^{6}$	78.07(2)	Se3	Ba1	$Se3^{9}$	127.46(3)
Se3	Cs1	$Se6^{12}$	137.25(3)	Se1	Ba1	Se3	57.94(4)
Se3	Cs1	$Se3^{10}$	69.78(2)	Cl1	Ba1	$Se1^{9}$	70.09(4)
Se3	Cs1	$Se5^{6}$	144.55(3)	Cl1	Ba1	$Se1^{10}$	142.34(4)
$Se5^{7}$	Cs1	$Se6^{12}$	86.64(3)	Cl1	Ba1	Se2	135.93(4)
$Se5^{7}$	Cs1	Se3	103.16(3)	Cl1	Ba1	$Se2^{4}$	74.80(4)
$Se5^{6}$	Cs1	$Se3^{10}$	92.33(3)	Cl1	Ba1	$Se3^{10}$	82.48(4)
$Se5^{7}$	Cs1	$Se3^{10}$	139.58(3)	Cl1	Ba1	Se3	81.97(4)
$Se5^{7}$	Cs1	$Se5^{6}$	70.400(19)	Cl1	Ba1	$Se3^{9}$	121.48(4)
Cl1	Cs1	$Se1^{11}$	144.95(4)	Se4	Ba2	$Se4^{6}$	97.10(2)
Cl1	Cs1	$Se6^{12}$	147.90(4)	Se4	Ba2	$Se6^{7}$	152.86(3)
$Cl1^{10}$	Cs1	$Se6^{12}$	61.55(4)	$Se4^{6}$	Ba2	$Se6^{7}$	71.83(2)
$Cl1^{10}$	Cs1	$Se3^{10}$	66.35(4)	Se4	Ba2	$Se6^{2}$	93.45(2)
Cl1	Cs1	$Se3^{10}$	69.24(4)	Se4	Ba2	$Se5^{7}$	143.95(3)
$Cl1^{10}$	Cs1	Se3	126.63(4)	$Se4^{6}$	Ba2	$Se5^{6}$	60.03(2)
Cl1	Cs1	Se3	72.01(4)	Se4	Ba2	Se5	57.61(2)
$Cl1^{10}$	Cs1	$Se5^{7}$	129.95(4)	$Se4^{6}$	Ba2	Se5	70.52(2)
Cl1	Cs1	$Se5^{7}$	70.78(4)	Se4	Ba2	$Se5^{6}$	74.47(2)
Cl1	Cs1	$Se5^{6}$	73.07(4)	$Se6^{2}$	Ba2	$Se4^{6}$	142.49(3)
$Cl1^{10}$	Cs1	$Se5^{6}$	66.08(4)	$Se6^{2}$	Ba2	$Se6^{7}$	82.57(2)
Cl1	Cs1	$Cl1^{10}$	116.56(5)	$Se6^{2}$	Ba2	$Se5^{6}$	156.84(3)
$Se1^{9}$	Ba1	$Se1^{10}$	145.31(2)	$Se6^{7}$	Ba2	$Se5^{6}$	117.12(3)
$Se1^{9}$	Ba1	$Se2^{4}$	93.74(2)	$Se6^{7}$	Ba2	Se5	95.26(2)
$Se1^{10}$	Ba1	$Se2^{4}$	87.25(2)	$Se6^{2}$	Ba2	Se5	85.47(2)
$Se1^{9}$	Ba1	$Se3^{9}$	58.09(2)	$Se5^{7}$	Ba2	$Se6^{2}$	96.37(3)
$Se1^{9}$	Ba1	$Se3^{10}$	151.83(3)	$Se5^{7}$	Ba2	$Se6^{7}$	63.09(2)
$Se1^{10}$	Ba1	Se3	99.26(3)	$Se5^{6}$	Ba2	Se5	103.55(2)
$Se1^{10}$	Ba1	$Se3^{10}$	60.39(2)	$Se5^{7}$	Ba2	$Se5^{6}$	83.364(14)
$Se1^{9}$	Ba1	Se3	97.56(3)	$Se5^{7}$	Ba2	Se5	157.662(17)
$Se1^{10}$	Ba1	$Se3^{9}$	87.76(2)	Cl1	Ba2	Se4	70.25(4)
Se2	Ba1	$Se1^{9}$	88.12(2)	Cl1	Ba2	$Se4^{6}$	145.37(4)
Se2	Ba1	$Se1^{10}$	73.14(2)	Cl1	Ba2	$Se6^{7}$	132.18(4)
Se2	Ba1	$Se2^{4}$	146.698(18)	Cl1	Ba2	$Se6^{2}$	71.85(4)
Se2	Ba1	Se3	62.92(2)	Cl1	Ba2	$Se5^{7}$	80.08(4)

Cl1	Ba2	Se5	121.26(4)	Se6	Ba3	Se1[3]	161.10(3)
Cl1	Ba2	Se5[6]	85.36(4)	Se6	Ba3	Se1[4]	72.44(2)
Se4	Ba3	Se4[1]	109.12(3)	Se6	Ba3	Se6[1]	102.06(3)
Se4	Ba3	Se1[3]	101.57(2)	Se6	Ba3	Se2[4]	101.47(2)
Se4[1]	Ba3	Se1[4]	133.38(3)	Se6	Ba3	Cl1[1]	68.44(4)
Se4[1]	Ba3	Se1[3]	97.50(2)	Se2[3]	Ba3	Se4[1]	74.00(2)
Se4	Ba3	Se1[4]	99.23(2)	Se2[3]	Ba3	Se1[4]	91.58(2)
Se4[1]	Ba3	Se6	90.74(2)	Se2[3]	Ba3	Se1[3]	58.56(2)
Se4[1]	Ba3	Se6[1]	56.90(2)	Se2[3]	Ba3	Se6	140.33(3)
Se4	Ba3	Se6	59.58(2)	Se2[4]	Ba3	Se6[1]	113.64(2)
Se4	Ba3	Se6[1]	68.26(2)	Se2[3]	Ba3	Se6[1]	99.59(2)
Se4[1]	Ba3	Se2[4]	166.29(3)	Se2[3]	Ba3	Se2[4]	99.66(3)
Se4	Ba3	Se2[4]	72.68(2)	Se2[3]	Ba3	Cl1[1]	71.89(4)
Se4	Ba3	Se2[3]	159.98(3)	Cl1[1]	Ba3	Se1[3]	130.45(4)
Se4[1]	Ba3	Cl1[1]	66.95(4)	Cl1[1]	Ba3	Se6[1]	123.05(4)
Se4	Ba3	Cl1[1]	127.93(4)	Cl1[1]	Ba3	Se2[4]	123.31(4)
Se1[4]	Ba3	Se1[3]	112.41(3)	Se2	B1	Se1	115.2(5)
Se1[4]	Ba3	Se6[1]	167.17(2)	Se3	B1	Se1	120.1(5)
Se1[3]	Ba3	Se6[1]	69.12(2)	Se3	B1	Se2	124.6(5)
Se1[4]	Ba3	Se2[4]	57.71(2)	Se4	B2	Se6	116.2(5)
Se1[3]	Ba3	Se2[4]	69.00(2)	Se5	B2	Se4	119.2(5)
Se1[4]	Ba3	Cl1[1]	66.43(4)	Se5	B2	Se6	124.6(5)

[1]3/2-X,-1/2+Y,+Z; [2]3/2-X,1/2+Y,+Z; [3]1/2+X,-1+Y,1/2-Z; [4]1-X,-1/2+Y,1/2-Z; [5]1/2-X,1/2+Y,+Z; [6]1-X,1-Y,1-Z; [7]-1/2+X,3/2-Y,1-Z; [8]1-X,1/2+Y,1/2-Z; [9]1/2+X,+Y,1/2-Z; [10]1/2-X,-1/2+Y,+Z; [11]-X,-1/2+Y,1/2-Z; [12]-1+X,+Y,+Z; [13]-1/2+X,1+Y,1/2-Z; [14]-X,1/2+Y,1/2-Z; [15]-1/2+X,+Y,1/2-Z; [16]1+X,+Y,+Z; [17]1/2+X,3/2-Y,1-Z

Table S25. Selected bond angles [°] for $CsBa_3(BSe_3)_2Br$.

Atom	Atom	Atom	Angle/°	Atom	Atom	Atom	Angle/°
Se1^{12}	Cs1	Se1^{11}	65.41(5)	Se1	Ba1	Se2^{1}	136.69(5)
Se2^{10}	Cs1	Se1^{11}	60.58(4)	Se1^{5}	Ba1	Se2^{4}	136.69(5)
Se2^{13}	Cs1	Se1^{11}	94.22(4)	Se1	Ba1	Se2^{5}	100.31(5)
Se2^{13}	Cs1	Se1^{12}	60.58(4)	Se1^{5}	Ba1	Se2^{1}	89.94(4)
Se2^{10}	Cs1	Se1^{12}	94.22(4)	Se1^{5}	Ba1	Se2^{5}	58.18(4)
Se2^{13}	Cs1	Se2^{10}	63.03(5)	Se1	Ba1	Br1^{6}	68.54(4)
Se2^{13}	Cs1	Br1^{14}	119.58(6)	Se1^{5}	Ba1	Br1^{6}	68.54(4)
Se2^{10}	Cs1	Br1^{14}	119.58(6)	Se1^{4}	Ba1	Br1^{6}	125.48(4)
Br1	Cs1	Se1^{11}	139.87(4)	Se1^{1}	Ba1	Br1^{6}	125.48(4)
Br1	Cs1	Se1^{12}	139.87(4)	Se2^{1}	Ba1	Se1^{4}	100.10(5)
Br1^{14}	Cs1	Se1^{11}	59.04(4)	Se2^{1}	Ba1	Se1^{1}	58.15(4)
Br1^{14}	Cs1	Se1^{12}	59.04(4)	Se2^{4}	Ba1	Se1^{4}	58.15(4)
Br1	Cs1	Se2^{10}	124.48(7)	Se2^{4}	Ba1	Se1^{1}	100.10(5)
Br1	Cs1	Se2^{13}	124.48(7)	Se2^{1}	Ba1	Se2^{5}	110.65(5)
Br1	Cs1	Br1^{14}	103.01(7)	Se2^{4}	Ba1	Se2	110.65(5)
Se3^{15}	Cs1	Se1^{11}	122.53(7)	Se2	Ba1	Se2^{5}	65.71(5)
Se3	Cs1	Se1^{11}	63.99(4)	Se2^{1}	Ba1	Se2^{4}	68.08(6)
Se3	Cs1	Se1^{12}	122.54(7)	Se2^{1}	Ba1	Se2	163.77(5)
Se3^{15}	Cs1	Se1^{12}	63.99(4)	Se2^{4}	Ba1	Se2^{5}	163.77(5)
Se3	Cs1	Se2^{13}	146.43(5)	Se2^{4}	Ba1	Br1^{6}	68.77(4)
Se3^{15}	Cs1	Se2^{10}	146.43(5)	Se2^{1}	Ba1	Br1^{6}	68.77(4)
Se3	Cs1	Se2^{10}	83.61(4)	Br1^{6}	Ba1	Se2^{5}	126.69(5)
Se3^{15}	Cs1	Se2^{13}	83.61(4)	Br1^{6}	Ba1	Se2	126.69(5)
Se3^{15}	Cs1	Br1^{14}	72.55(5)	Se1^{7}	Ba2	Se1	148.80(3)
Se3	Cs1	Br1	76.65(4)	Se1^{7}	Ba2	Se2^{8}	90.70(4)
Se3	Cs1	Br1^{14}	72.55(5)	Se1^{7}	Ba2	Br1	69.86(5)
Se3^{15}	Cs1	Br1	76.65(4)	Se1	Ba2	Br1	139.82(5)
Se3^{15}	Cs1	Se3	129.23(8)	Se1^{7}	Ba2	Se3^{9}	83.67(4)
Se1^{5}	Ba1	Se1^{1}	98.53(4)	Se1	Ba2	Se3^{8}	114.48(4)
Se1	Ba1	Se1^{5}	81.22(6)	Se1^{7}	Ba2	Se3^{8}	73.41(4)
Se1	Ba1	Se1^{4}	98.53(4)	Se1	Ba2	Se3^{9}	71.44(4)
Se1^{5}	Ba1	Se1^{4}	165.00(4)	Se2^{9}	Ba2	Se1^{7}	91.80(4)
Se1	Ba1	Se1^{1}	165.00(4)	Se2^{9}	Ba2	Se1	91.21(4)
Se1^{4}	Ba1	Se1^{1}	77.81(6)	Se2^{8}	Ba2	Se1	70.66(4)
Se1^{1}	Ba1	Se2^{5}	67.57(4)	Se2^{9}	Ba2	Se2^{8}	146.98(3)
Se1^{1}	Ba1	Se2	107.46(5)	Se2^{9}	Ba2	Br1	71.55(5)
Se1^{5}	Ba1	Se2	100.31(5)	Se2^{8}	Ba2	Br1	139.23(5)
Se1^{4}	Ba1	Se2	67.57(4)	Se2^{8}	Ba2	Se3^{9}	88.58(4)
Se1^{4}	Ba1	Se2^{5}	107.46(5)	Se2^{8}	Ba2	Se3^{8}	59.98(4)
Se1	Ba1	Se2	58.18(4)	Se2^{9}	Ba2	Se3^{8}	151.00(4)
Se1	Ba1	Se2^{4}	89.94(4)	Se2^{9}	Ba2	Se3^{9}	59.04(4)

Br1	Ba2	Se3[9]	122.59(5)	Se3	Ba2	Se3[8]	84.23(2)
Se3	Ba2	Se1	62.34(4)	Se3[8]	Ba2	Se3[9]	139.94(5)
Se3	Ba2	Se1[7]	147.60(5)	Se3	Ba2	Se3[9]	127.40(5)
Se3	Ba2	Se2[9]	97.12(4)	Se2	B1	Se1	116.3(8)
Se3	Ba2	Se2[8]	98.21(4)	Se3	B1	Se1	123.3(8)
Se3	Ba2	Br1	83.54(5)	Se3	B1	Se2	120.3(7)
Se3[8]	Ba2	Br1	79.88(5)				

[1]1-X,1-Y,1/2+Z; [2]1-X,1-Y,-1/2+Z; [3]1/2+X,3/2-Y,1/2+Z; [4]+X,1-Y,1/2+Z; [5]1-X,+Y,+Z; [6]1/2-X,3/2-Y,1/2+Z; [7]1/2-X,3/2-Y,-1/2+Z; [8]+X,1-Y,-1/2+Z; [9]1/2-X,1/2+Y,+Z; [10]1/2-X,1/2-Y,-1/2+Z; [11]1/2-X,-1/2+Y,+Z; [12]-1/2+X,-1/2+Y,+Z; [13]-1/2+X,1/2-Y,-1/2+Z; [14]-X,1-Y,1/2+Z; [15]-X,+Y,+Z; [16]1/2+X,1/2+Y,+Z; [17]1/2-X,1/2-Y,1/2+Z; [18]-X,1-Y,-1/2+Z

Table S26. Selected bond angles [°] for $CsBa_3(BSe_3)_2I$.

Atom	Atom	Atom	Angle/°	Atom	Atom	Atom	Angle/°
$I1^{12}$	Cs1	$I1^{3}$	98.22(5)	Se1	Ba1	$Se2^{4}$	100.66(5)
$I1^{12}$	Cs1	$Se1^{4}$	137.71(4)	Se1	Ba1	Se2	57.84(4)
$I1^{12}$	Cs1	Se1	137.71(4)	$Se1^{4}$	Ba1	$Se2^{4}$	57.84(4)
$I1^{3}$	Cs1	$Se1^{4}$	60.95(4)	$Se1^{4}$	Ba1	$Se2^{5}$	135.71(5)
$I1^{3}$	Cs1	Se1	60.95(4)	Se1	Ba1	$Se2^{5}$	88.36(4)
$I1^{12}$	Cs1	$Se3^{7}$	74.08(4)	$Se1^{5}$	Ba1	Se2	68.26(4)
$I1^{12}$	Cs1	$Se3^{12}$	74.08(4)	$Se1^{1}$	Ba1	Se2	108.81(5)
$Se1^{4}$	Cs1	Se1	67.11(6)	$Se1^{5}$	Ba1	$Se2^{4}$	108.81(5)
$Se2^{2}$	Cs1	$I1^{3}$	123.35(5)	$Se1^{4}$	Ba1	$Se2^{1}$	88.36(4)
$Se2^{2}$	Cs1	$I1^{12}$	123.64(6)	Se2	Ba1	$I1^{3}$	126.32(4)
$Se2^{8}$	Cs1	$I1^{12}$	123.64(6)	$Se2^{4}$	Ba1	$I1^{3}$	126.32(4)
$Se2^{8}$	Cs1	$I1^{3}$	123.35(5)	$Se2^{1}$	Ba1	$I1^{3}$	67.69(4)
$Se2^{2}$	Cs1	Se1	97.55(5)	$Se2^{5}$	Ba1	$I1^{3}$	67.69(4)
$Se2^{8}$	Cs1	$Se1^{4}$	97.55(5)	$Se2^{1}$	Ba1	$Se1^{5}$	100.91(5)
$Se2^{8}$	Cs1	Se1	62.40(4)	$Se2^{5}$	Ba1	$Se1^{5}$	58.02(4)
$Se2^{2}$	Cs1	$Se1^{4}$	62.40(4)	$Se2^{5}$	Ba1	$Se1^{1}$	100.91(5)
$Se2^{2}$	Cs1	$Se2^{8}$	65.09(6)	$Se2^{1}$	Ba1	$Se1^{1}$	58.02(4)
$Se2^{8}$	Cs1	$Se3^{7}$	84.25(4)	Se2	Ba1	$Se2^{4}$	66.47(6)
$Se2^{2}$	Cs1	$Se3^{7}$	149.30(6)	$Se2^{1}$	Ba1	Se2	165.35(5)
$Se2^{2}$	Cs1	$Se3^{12}$	84.25(4)	$Se2^{5}$	Ba1	Se2	110.13(5)
$Se2^{8}$	Cs1	$Se3^{12}$	149.30(6)	$Se2^{5}$	Ba1	$Se2^{4}$	165.35(5)
$Se3^{7}$	Cs1	$I1^{3}$	71.60(5)	$Se2^{1}$	Ba1	$Se2^{4}$	110.13(5)
$Se3^{12}$	Cs1	$I1^{3}$	71.60(5)	$Se2^{1}$	Ba1	$Se2^{5}$	69.32(6)
$Se3^{7}$	Cs1	$Se1^{4}$	123.96(7)	$Se1^{6}$	Ba2	I1	69.77(4)
$Se3^{12}$	Cs1	$Se1^{4}$	64.67(4)	Se1	Ba2	I1	137.50(5)
$Se3^{7}$	Cs1	Se1	64.67(4)	Se1	Ba2	$Se1^{6}$	151.25(3)
$Se3^{12}$	Cs1	Se1	123.96(7)	$Se1^{6}$	Ba2	$Se3^{7}$	83.58(5)
$Se3^{7}$	Cs1	$Se3^{12}$	126.33(8)	Se1	Ba2	$Se3^{8}$	114.66(5)
$Se1^{4}$	Ba1	$I1^{3}$	68.51(4)	Se1	Ba2	$Se3^{7}$	73.27(4)
Se1	Ba1	$I1^{3}$	68.51(4)	$Se1^{6}$	Ba2	$Se3^{8}$	74.09(5)
$Se1^{1}$	Ba1	$I1^{3}$	124.55(4)	$Se2^{8}$	Ba2	I1	137.89(5)
$Se1^{5}$	Ba1	$I1^{3}$	124.55(4)	$Se2^{7}$	Ba2	I1	70.27(4)
$Se1^{1}$	Ba1	$Se1^{5}$	78.40(6)	$Se2^{7}$	Ba2	Se1	92.91(5)
Se1	Ba1	$Se1^{1}$	166.10(5)	$Se2^{8}$	Ba2	Se1	72.04(4)
$Se1^{4}$	Ba1	$Se1^{5}$	166.10(5)	$Se2^{8}$	Ba2	$Se1^{6}$	92.03(5)
$Se1^{4}$	Ba1	Se1	81.76(7)	$Se2^{7}$	Ba2	$Se1^{6}$	89.42(4)
Se1	Ba1	$Se1^{5}$	98.25(5)	$Se2^{7}$	Ba2	$Se2^{8}$	149.75(4)
$Se1^{4}$	Ba1	$Se1^{1}$	98.25(5)	$Se2^{7}$	Ba2	$Se3^{7}$	59.02(4)
$Se1^{1}$	Ba1	$Se2^{4}$	68.26(4)	$Se2^{8}$	Ba2	$Se3^{8}$	59.76(5)
Se1	Ba1	$Se2^{1}$	135.71(5)	$Se2^{7}$	Ba2	$Se3^{8}$	148.24(5)
$Se1^{4}$	Ba1	Se2	100.66(5)	$Se2^{8}$	Ba2	$Se3^{7}$	91.12(4)

Se3	Ba2	I1	80.66(4)	Se3	Ba2	Se3[7]	128.50(6)
Se3[7]	Ba2	I1	122.38(5)	Se3	Ba2	Se3[8]	83.24(3)
Se3[8]	Ba2	I1	78.52(5)	Se3[8]	Ba2	Se3[7]	141.74(5)
Se3	Ba2	Se1	62.46(4)	Se1	B1	Se2	116.7(9)
Se3	Ba2	Se1[6]	145.55(5)	Se3	B1	Se1	124.1(9)
Se3	Ba2	Se2[7]	97.06(5)	Se3	B1	Se2	119.2(9)
Se3	Ba2	Se2[8]	98.65(5)				

[1]1-X,1-Y,-1/2+Z; [2]1-X,1-Y,1/2+Z; [3]3/2-X,3/2-Y,-1/2+Z; [4]1-X,+Y,+Z; [5]+X,1-Y,-1/2+Z; [6]3/2-X,3/2-Y,1/2+Z; [7]3/2-X,1/2+Y,+Z; [8]+X,1-Y,1/2+Z; [9]2-X,+Y,+Z; [10]1/2+X,-1/2+Y,+Z; [11]1-X,2-Y,1/2+Z; [12]-1/2+X,1/2+Y,+Z; [13]3/2-X,-1/2+Y,+Z

Table S27. Experimental bandgaps and HSE06 bandgaps of title compounds.

Formula	Bandgap (Experiment)	Bandgap (HSE06)
$RbBa_3(BSe_3)_2Cl$	2.94 eV	3.01 eV
$RbBa_3(BSe_3)_2Br$	2.92 eV	2.99 eV
$RbBa_3(BSe_3)_2I$	3.00 eV	3.03 eV
$CsBa_3(BSe_3)_2Cl$	3.00 eV	3.02 eV
$CsBa_3(BSe_3)_2Br$	3.04 eV	3.04 eV
$CsBa_3(BSe_3)_2I$	3.02 eV	3.08 eV

Table S28. Measured LIDTs of $CsBa_3(BSe_3)_2I$ and benchmark $AgGaS_2$.

Formula	Damage energy (mJ)	Spot area (cm^2)	τ_p (ns)	LIDT ($MW\cdot cm^{-2}$)
$CsBa_3(BSe_3)_2I$	8.14	0.017	50	9.58
$AgGaS_2$	1.56	0.017	50	1.83

Table S29. Summary LIDTs of related compounds.

Formula	Space group	LIDT (× AGS)	Ref.
$BaLi_2GeSe_4$	$I\bar{4}2m$	1.0	[15]
$BaLi_2SnSe_4$	$I\bar{4}2m$	1.0	[15]
$NaGa_3Se_5$	$P2_12_12_1$	1.7	[16]
$PbGa_2GeSe_6$	$Fdd2$	3.7	[17]
$Na_2Hg_3Ge_2S_8$	$P\bar{4}c2$	3.0	[18]
$Na_2Hg_3Sn_2S_8$	$P\bar{4}c2$	1.0	[18]
$Na_2ZnSn_2S_6$	$Fdd2$	2.0	[19]
$CsBa_3(BSe_3)_2I$	$Cmc2_1$	5.2	This work

Table S30. Summary of selected asymmetric chalcogenides.

Label	Formula	Space group	Bandgap (eV)	SHG (× AGS)	Ref.
1	$CsCd_4In_5S_{12}$	*R*3	2.47	1.1	[20]
2	$KAg_3Ga_8Se_{14}$	*Cm*	2.27	0.6	[21]
3	$BaAg_2SnS_4$	*I*222	1.77	0.4	[22]
4	$Na_2In_2SiS_6$	*Cc*	2.47	0.3	[23]
5	β-$PbGa_2S_4$	$Pna2_1$	2.46	0.1	[24]
6	$Ba_6Zn_7Ga_2S_{16}$	*R3*	3.50	0.5	[25]
7	$SrLi_2GeS_4$	$I\bar{4}2m$	3.75	0.5	[26]
/	$RbBa_3(BSe_3)_2Cl$	$Cmc2_1$	2.94	0.9	This work
/	$RbBa_3(BSe_3)_2Br$	$Cmc2_1$	2.92	0.9	This work
/	$RbBa_3(BSe_3)_2I$	$Cmc2_1$	3.00	1.0	This work
/	$CsBa_3(BSe_3)_2Br$	$Cmc2_1$	3.04	1.0	This work
/	$CsBa_3(BSe_3)_2I$	$Cmc2_1$	3.02	1.0	This work

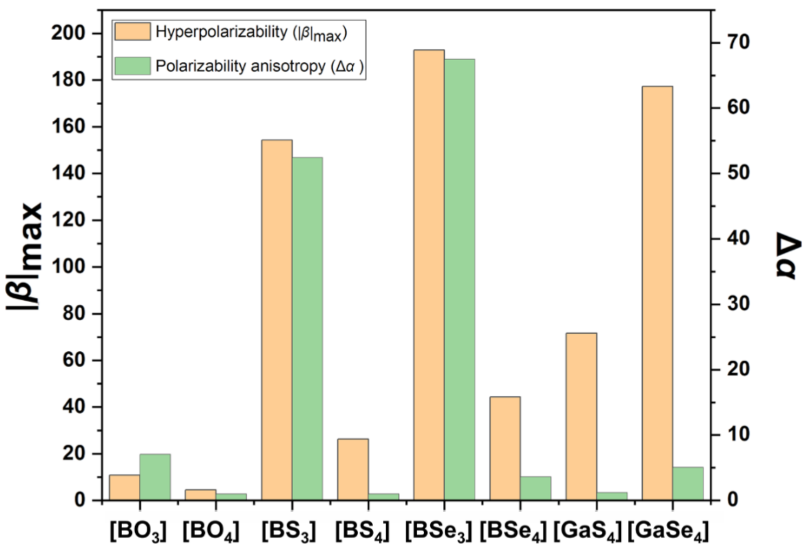

Figure S1. The comparison of microstructural performances of several typical units.

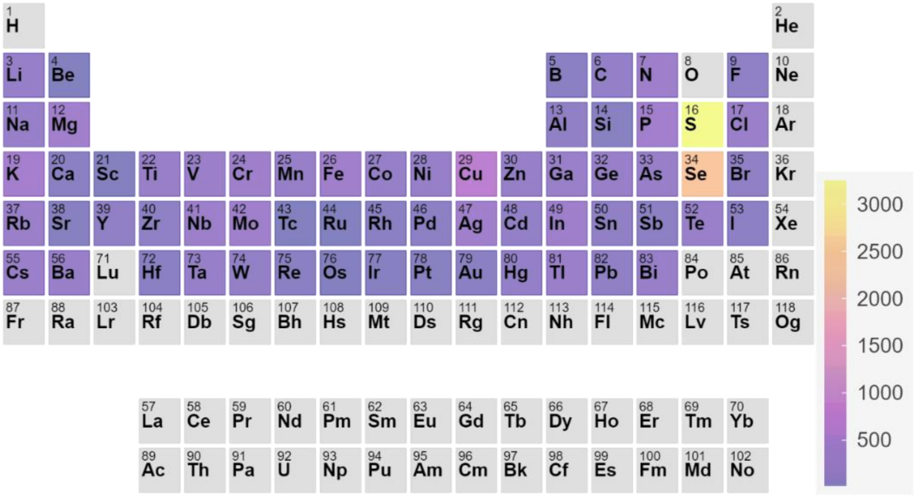

Figure S2. Elements of the periodic table contained in the dataset.

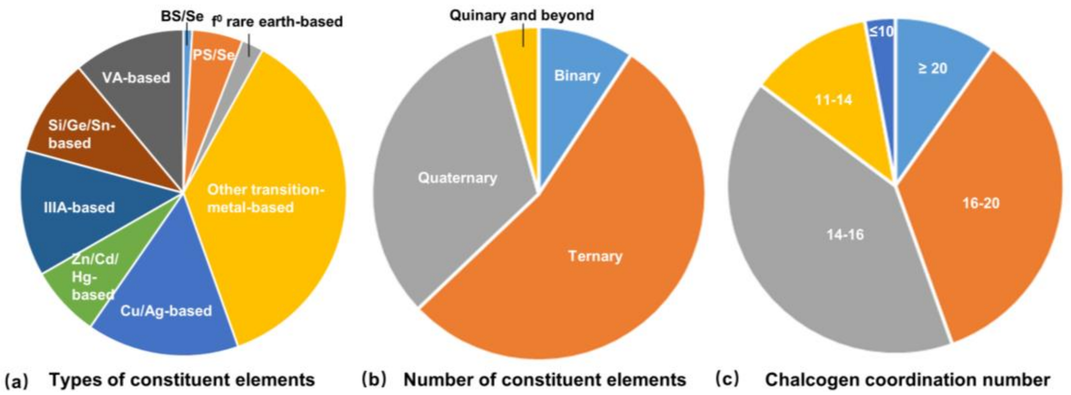

Figure S3. Distribution of chalcogenides in the dataset.

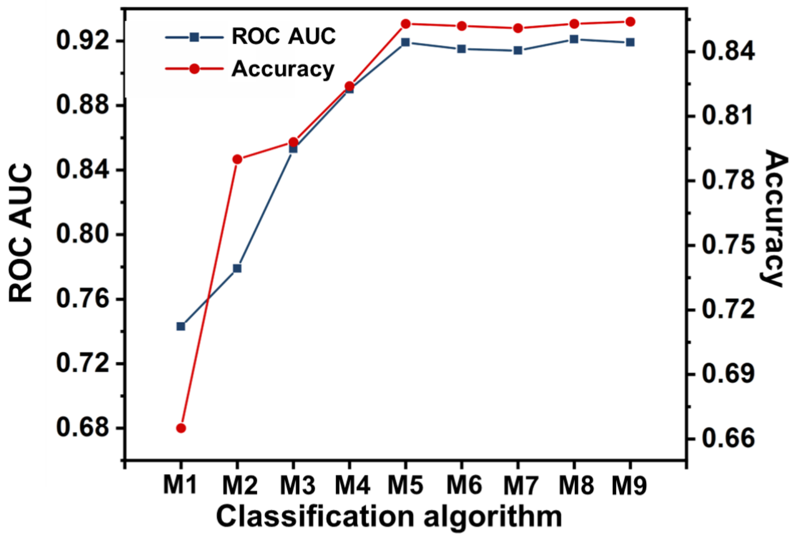

Figure S4. Comparison of performance of selected classification models.

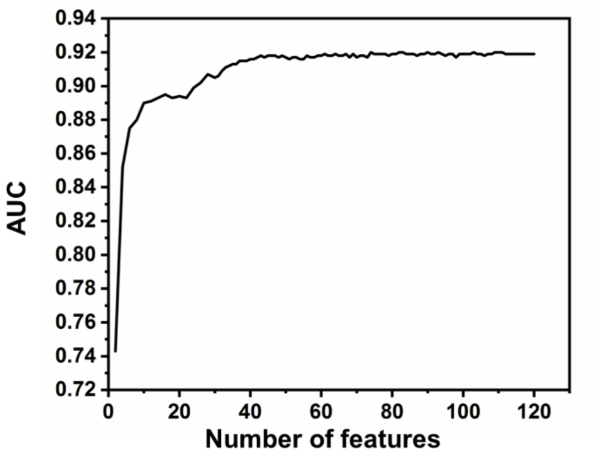

Figure S5. Model performance with different number of features.

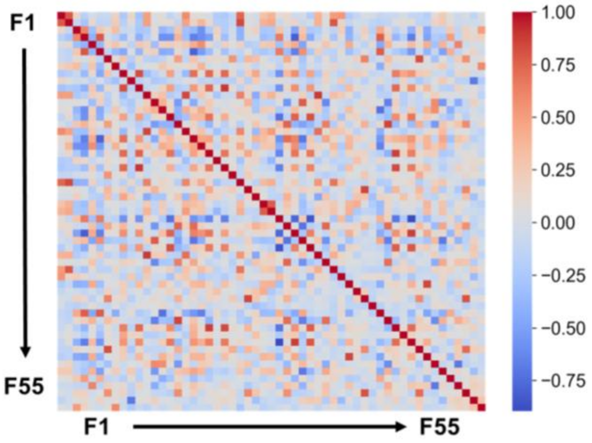

Figure S6. Heat map of the Pearson correlation coefficient matrix among the selected 55 features.

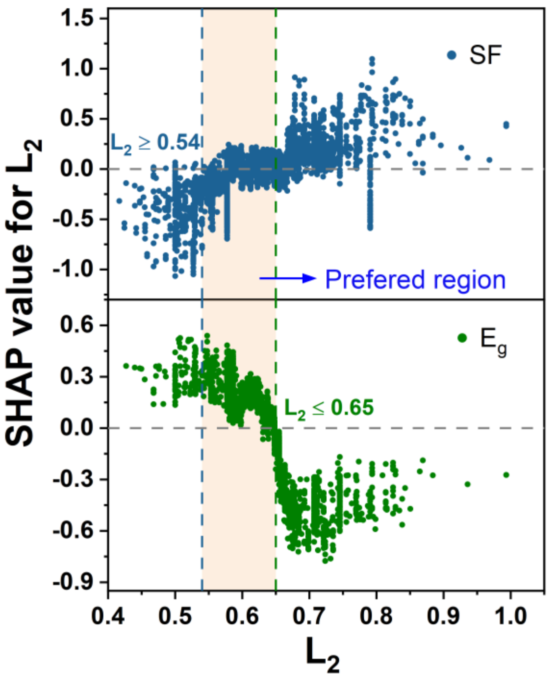

Figure S7. SHAP values for L_2 in the two classifiers, showing the specific effects on SF and E_g, respectively.

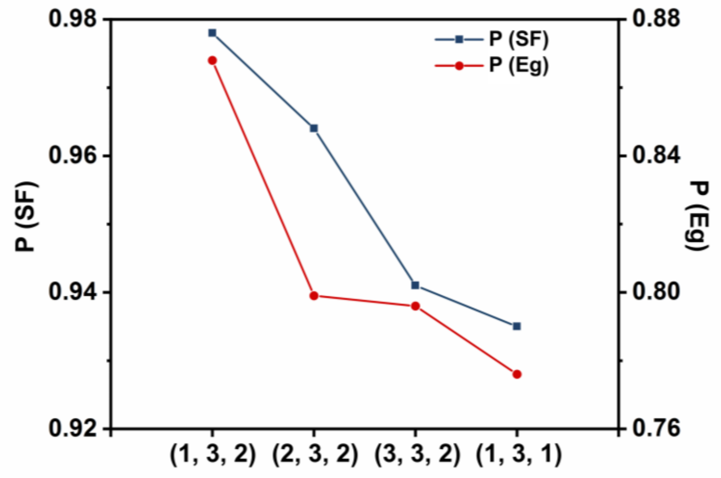

Figure S8. Comparison of the average performances of ML-predicted four top precursor ratios.

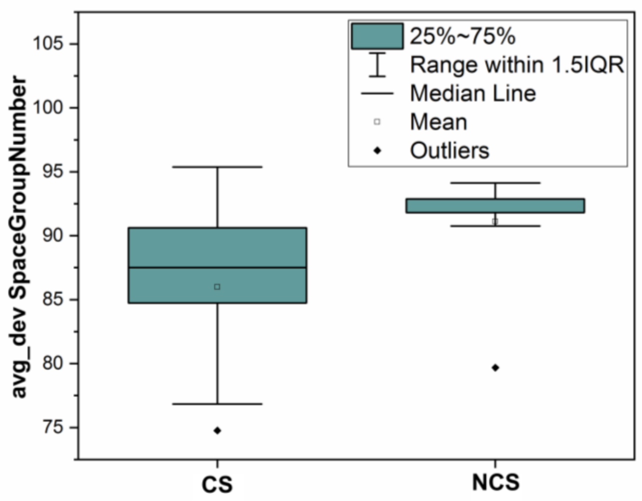

Figure S9. Distribution of the average deviation of space group number for NCS and CS structures in the reported selenoborates.

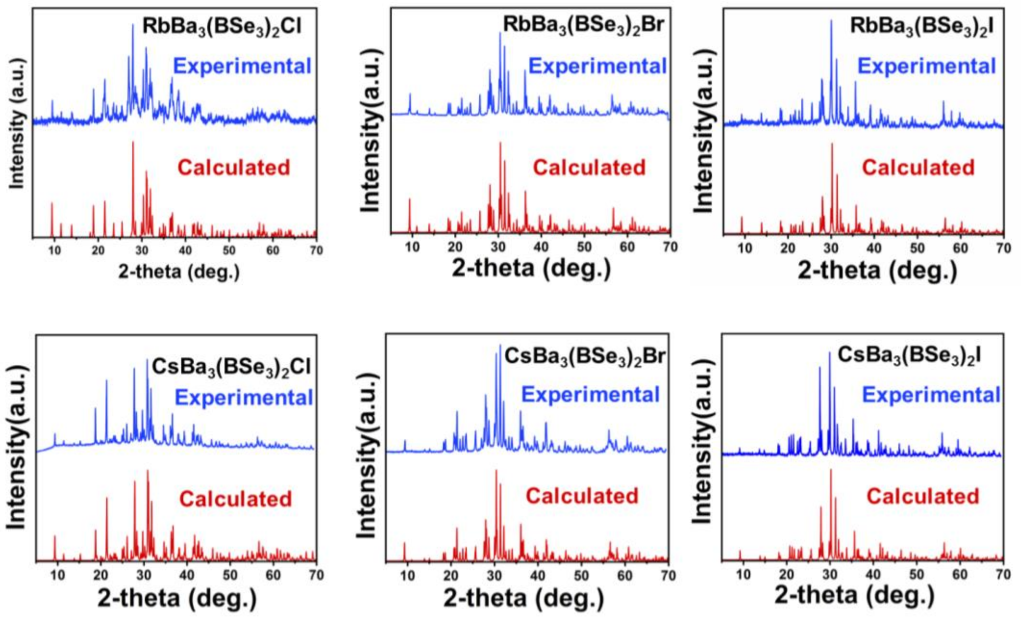

Figure S10. Experimental and theoretical X-ray diffraction patterns of $ABa_3(BSe_3)_2X$ (A= Rb, Cs; X=Cl, Br, I).

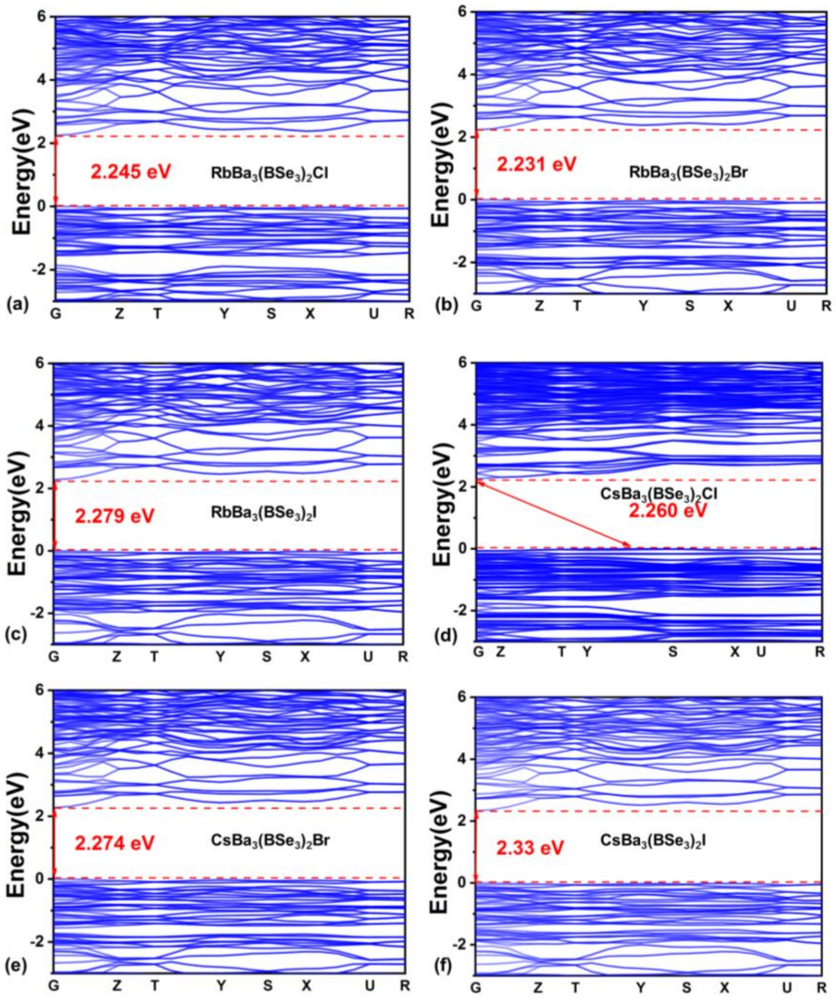

Figure S11. Band structures of $ABa_3(BSe_3)_2X$ (A= Rb, Cs; X=Cl, Br, I).

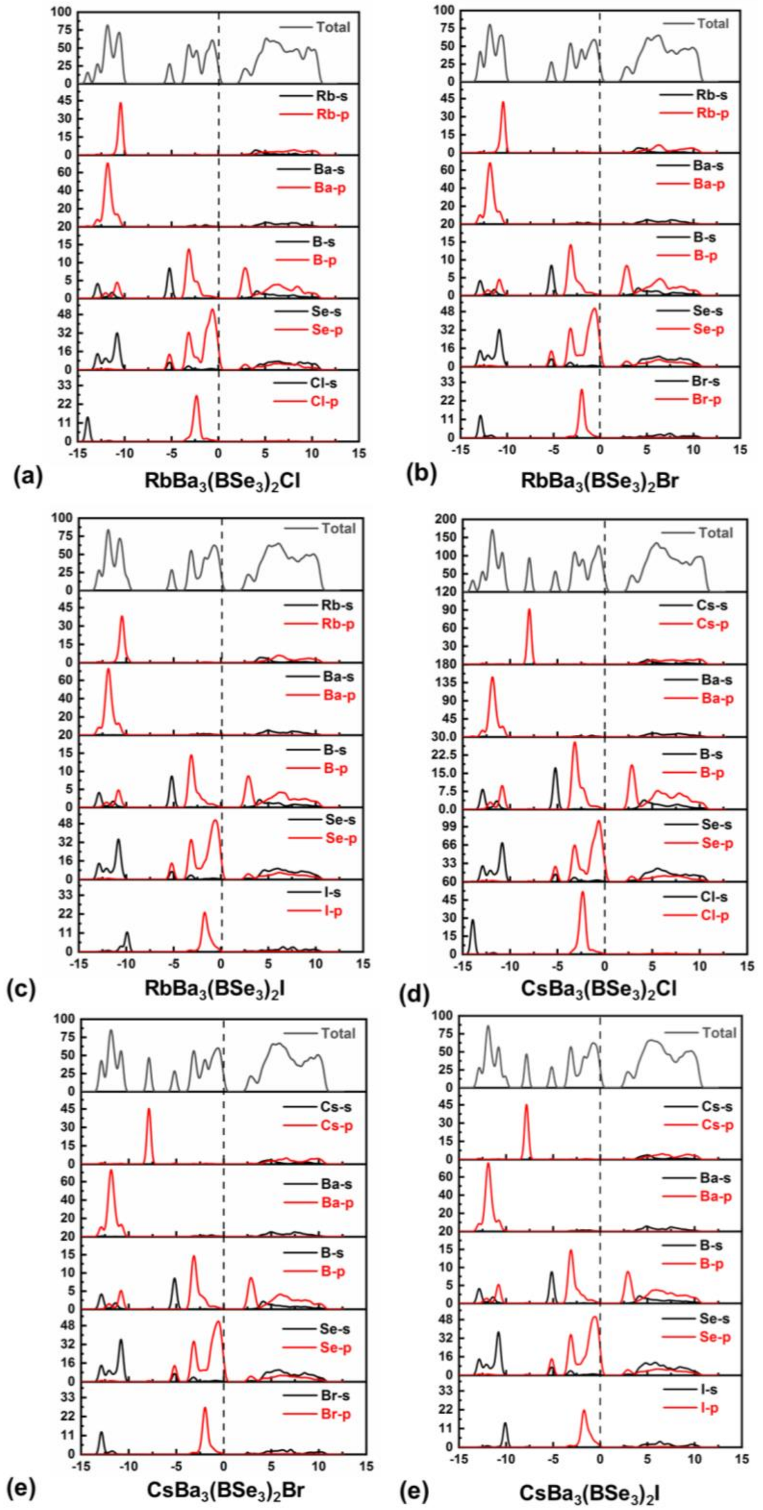

Figure S12. Density of states (DOS) of $ABa_3(BSe_3)_2X$ (A= Rb, Cs; X=Cl, Br, I).

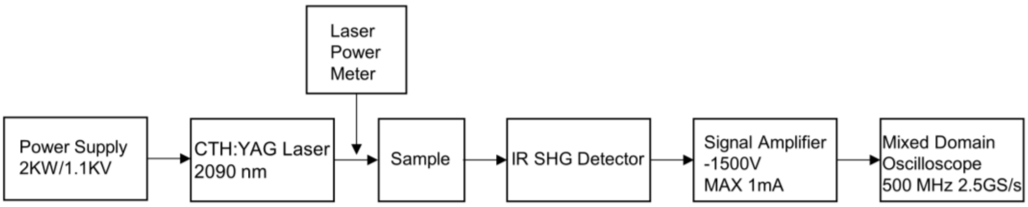

Figure S13. Experimental setup diagram for SHG.

Reference

[1] B. Krebs, W. Hamann, *J. Less-Common Metals* **1988**, *137*, 143-154.

[2] A. Hammerschmidt, C. Köster, J. Küper, A. Lindemann, B. Krebs, *Z. Anorg. Allg. Chem.* **2001**, *627*, 1253-1258.

[3] Y. Y. Li, B. X. Li, G. Zhang, L. J. Zhou, H. Lin, J. N. Shen, C. Y. Zhang, L. Chen, L. M. Wu, *Inorg. Chem.* **2015**, *54*, 4761-4767.

[4] M. Döch, A. Hammerschmidt, S. Pütz, B. P. D. J. P. Krebs, Sulfur,, Silicon, t. R. Elements, *Phosphorus, Sulfur Silicon Relat. Elem.* **2004**, *179*, 933 - 935.

[5] A. Hammerschmidt, J. Küper, L. Stork, B. Krebs, *Z. Anorg. Allg. Chem.* **1994**, *620*, 1898-1904.

[6] A. Lindemann, J. Küper, W. Hamann, J. Kuchinke, C. Köster, B. Krebs, *J. Solid State Chem.* **2001**, *157*, 206-212.

[7] A. Lindemann, J. Küper, C. Jansen, J. Kuchinke, C. Köster, A. Hammerschmidt, M. Döch, T. Pruß, B. Krebs, *Z. Anorg. Allg. Chem.* **2001**, *627*, 419-425.

[8] A. Hammerschmidt, A. Lindemann, M. Döch, C. Köster, B. Krebs, *Z. Anorg. Allg. Chem.* **2002**, *628*, 1561-1567.

[9] J. Kuchinke, A. Lindemann, C. Köster, A. Hammerschmidt, M. Döch, T. Pruss, B. Krebs, *Phosphorus, Sulfur Silicon Relat. Elem.* **2001**, *169*, 281-284.

[10] L. J. Chen, J. D. Liao, Y. J. Chuang, Y. S. Fu, *J. Am. Chem. Soc.* **2011**, *133*, 3704-3707.

[11] A. Lindemann, J. Kuchinke, C. Köster, A. Hammerschmidt, M. Döch, T. Pruss, B. Krebs, *Phosphorus, Sulfur Silicon Relat. Elem.* **2001**, *169*, 169-172.

[12] A. Hammerschmidt, A. Lindemann, M. Döch, B. Krebs, *Solid State Sci.* **2002**, *4*, 1449-1455.

[13] A. Hammerschmidt, M. Döch, S. Pütz, H. Eckert, B. Krebs, *Z. Anorg. Allg. Chem.* **2006**, *632*, 1219-1226.

[14] S. Pütz, M. Döch, A. Hammerschmidt, A. Lindemann, H. Eckert, T. Nilges, B. Krebs, *Solid State Sci.* **2006**, *8*, 764-772.

[15] K. Wu, B. Zhang, Z. Yang, S. Pan, *J. Am. Chem. Soc.* **2017**, *139*, 14885-14888.

[16] Q. T. Xu, S. S. Han, J. N. Li, S. P. Guo, *Inorg. Chem.* **2022**, *61*, 5479-5483.

[17] Z. Z. Luo, C. S. Lin, H. H. Cui, W. L. Zhang, H. Zhang, H. Chen, Z. Z. He, W. D. Cheng, *Chem. Mater.* **2015**, *27*, 914-922.

[18] K. Wu, Z. Yang, S. Pan, *Chem. Mater.* **2016**, *28*, 2795-2801.

[19] G. Li, K. Wu, Q. Liu, Z. Yang, S. Pan, *Sci. China Technol. Sci.* **2017**, *60*, 1465-1472.

[20] Y. Wang, R. Wang, X. Che, F. Liang, M. Luo, Y. Tang, Y. Cao, F. Huang, *J. Mater. Chem. C* **2022**, *10*, 5183-5189.

[21] J. N. Li, W. D. Yao, X. H. Li, W. Liu, H. G. Xue, S. P. Guo, *Chem. Commun.* **2021**, *57*, 1109-1112.

[22] H. Chen, P. F. Liu, B. X. Li, H. Lin, L. M. Wu, X. T. Wu, *Dalton Trans.* **2018**, *47*, 429-437.

[23] S. F. Li, B. W. Liu, M. J. Zhang, Y. H. Fan, H. Y. Zeng, G. C. Guo, *Inorg. Chem.* **2016**, *55*, 1480-1485.

[24]W. F. Chen, B. W. Liu, X. M. Jiang, G. C. Guo, *J. Alloys Compd.* **2022**, *905*, 164090.
[25] Y. Y. Li, P. F. Liu, L. M. Wu, *Chem. Mater.* **2017**, *29*, 5259-5266.
[26] Y. Yang, K. Wu, X. Wu, B. Zhang, L. Gao, *J. Mater. Chem. C* **2020**, *8*, 1762-1767.
